# Differential expression of microRNAs in response to *Papaya ringspot virus* infection in differentially responding genotypes of papaya (*Carica papaya* L.) and its wild relative

**DOI:** 10.3389/fpls.2024.1398437

**Published:** 2024-06-20

**Authors:** Basavaprabhu L. Patil, Savarni Tripathi

**Affiliations:** ^1^ ICAR-Indian Institute of Horticultural Research, Bengaluru, India; ^2^ ICAR-Indian Agricultural Research Institute, Regional Station, Pune, India

**Keywords:** papaya, virus, PRSV, resistance, miRNA, small RNA, differential expression

## Abstract

Papaya ringspot virus (PRSV) is one of the most devastating viruses of papaya that has significantly hampered papaya production across the globe. Although PRSV resistance is known in some of its wild relatives, such as *Vasconcellea cauliflora* and in some of the improved papaya genotypes, the molecular basis of this resistance mechanism has not been studied and understood. Plant microRNAs are an important class of small RNAs that regulate the gene expression in several plant species against the invading plant pathogens. These miRNAs are known to manifest the expression of genes involved in resistance against plant pathogens, through modulation of the plant’s biochemistry and physiology. In this study we made an attempt to study the overall expression pattern of small RNAs and more specifically the miRNAs in different papaya genotypes from India, that exhibit varying levels of tolerance or resistance to PRSV. Our study found that the expression of some of the miRNAs was differentially regulated in these papaya genotypes and they had entirely different miRNA expression profile in healthy and PRSV infected symptomatic plants. This data may help in improvement of papaya cultivars for resistance against PRSV through new breeding initiatives or biotechnological approaches such as genome editing.

## Introduction

Papaya (*Carica papaya* L.), belonging to the family Caricaceae, is one of the economically most important nutraceutical fruit crops grown in tropical and subtropical regions of the world. Worldwide, the ring spot disease of papaya caused by the Papaya ringspot virus (PRSV) and transmitted by aphid vectors in a non-persistent manner, has been the most devastating threat to papaya cultivation (Gonsalves et al., 2010; Mishra et al., 2016). PRSV, with a single-stranded positive sense RNA genome of 10 Kb length, encoding for a polyprotein that is eventually cleaved into 10 distinct proteins, belongs to the family *Potyviridae* and genus *Potyvirus* (Tripathi et al., 2008). The ringspot disease of papaya is identified by distorted leaves with mosaic pattern, wet oil streaks on petioles, stunted growth and ring spots on the fruits at later stages (Tripathi et al., 2008; Mishra et al., 2016).

Visible success has not been achieved in managing the PRSV under field conditions in many papaya growing regions of the world, mainly due to lack of agronomically accepted PRSV resistant papaya cultivars. Hence, developing PRSV resistant papaya varieties, either through conventional breeding or through biotechnological interventions, with acceptable agronomic traits and quality fruits is a priority (Mishra et al., 2016). Successful implementation of such virus resistant breeding programs, largely depends on the identification and selection of superior papaya genotypes that are resistant to PRSV (Prakash and Singh, 2013). Singh et al. (2006) conducted the study to identify superior papaya varieties that are tolerant to viral diseases under north Indian climatic conditions, by evaluating several accessions of papaya comprising of both Indian and exotic cultivars, along with promising selections. In an effort to develop PRSV resistant papaya, ICAR-Indian Agricultural Research Institute, Regional Station, Pune (IARI, RS, Pune, India) has successfully developed four PRSV tolerant dioecious lines named Pune Selection-1 (PS-1), PS-2, PS-3 and PS-5, by selecting and sib mating from segregating population of a land race of papaya called as Madhubala. All the four PS lines (-1, 2, 3 and 5) proved superior in respect of fruit yield and PRSV tolerance as compared to other commercial papaya varieties, suggesting that these lines can be used as a source of PRSV tolerance (Datar et al., 2013; Sharma and Tripathi, 2019; Sharma et al., 2019).

During the course of evolution, plants have developed diverse resistance mechanisms against the invading viruses such as RNA silencing, immune receptor signaling, protein degradation, hormone mediated defense, innate antiviral immunity, translation repression, small RNA-mediated antiviral defense, dominant viral resistance genes, resistance to virus movement, autophagy, and cross protection (Loebenstein, 2009; Muthamilarasan and Prasad, 2013; Park et al., 2013; Sett et al., 2022, Calil and Fontes, 2017, Patil et al., 2021). The RNA silencing is mediated by various types of small RNAs such as siRNAs, microRNAs, tasiRNAs etc (Weiberg and Jin, 2015). Unlike animals, plants lack defense cells and rely on the capacity of every cell to distinguish and defend against the invaders. Micro RNA is a class of unique small endogenous, non-coding RNA that is involved in regulation of gene expression by binding to the target mRNA leading to mRNA cleavage, translational repression, mRNA de-adenylation or transcriptional silencing thereby controlling the expression of the translated product (Jones-Rhoades et al., 2006; Winter et al., 2012). The control for gene expression is based on complementary pairing at specific positions for the target (Rhoades et al., 2002). These microRNAs are responsible for specific temporal and spatial control of their gene targets. Depending on the different environmental conditions, they act as an alternative strategy for editing the transcriptome during various environmental stresses. The role of miRNAs in plant development and stress response is well established and also has a crucial role in plant-virus interactions (Liu et al., 2017). Some of the plant miRNA families are highly conserved through thousands of millions of years, whereas some of them evolved and diversified to become specific to certain plant species and their genotypes (Axtell and Bartel, 2005; Axtell et al., 2007; Grimson et al., 2008). With the advent of high-throughput sequencing, it has been possible to identify not only the conserved miRNAs, but also plant species-specific miRNAs (Fahlgren et al., 2007; Moxon et al., 2008). Several studies have implicated the role of microRNAs that respond to diverse biotic stresses in various plant species and also diverse plant genotypes of same plant species. The miRNA accumulation at various levels in response to virus infection has been reported by various studies (Bazzini et al., 2007; Naqvi et al., 2010; Khraiwesh et al., 2012; Kumar, 2014). The plant miRNAs and their corresponding target genes have been identified to be responsive to infection by diverse viruses such as cucumber green mottle mosaic virus (CGMMV), cowpea severe mosaic virus (CPSMV), mungbean yellow mosaic India virus (MYMIV), sugarcane mosaic virus (SCMV) and soybean mosaic virus (SMV) in cucumber, cowpea, common bean, black gram, corn and soybean (Yin et al., 2013; Kundu et al., 2017; Liang et al., 2019; Patwa et al., 2019; Martins et al., 2020).

Several Potyviruses are also reported to result in differential expression of miRNAs in the host plants. Tobacco etch virus (TEV) and Potato virus Y (PVY) representative of *Potyviridae* family show higher expression of miR166, miR171, miR159, and miR167 (Bazzini et al., 2007). Further the differential expression of miR160, miR169, miR164 and miR156 was observed in infected *Nicotiana benthamiana* leaf samples (Bazzini et al., 2007). Another study on PVY, reported higher expression of miR398, miR171, miR168, and miR156, while double infection of PVX-PVY showed synergistic effect on phenotype and resulted in higher expression of miR156 and miR398, while miR171 was downregulated (Pacheco et al., 2012). In spite of accumulation of certain miRNAs, the targets of the corresponding miRNA were upregulated, and SPL6-IV mRNA was suggested to be inhibited in PVX-PVY infection (Pacheco et al., 2012). TEV infection alone increases the expression of miR159 and miR168 in *N. benthamiana* (Várallyay et al., 2010).

Recent advances in the field of miRNA-mediated gene silencing have been reported to be applied in several agricultural crop species to counter diverse virus infections (Tiwari et al., 2014; Khalid et al., 2017). In order to understand the mechanism of PRSV tolerance in Pune Selection (PS-3) as compared to susceptible papaya genotypes (PM), in this study, miRNA-based regulatory network is investigated using high-throughput deep sequencing technology. For the first time we report here differential expression of microRNAs in response to PRSV infection in both PRSV tolerant and susceptible genotypes of papaya (*C. papaya*.).

## Materials and methods

### Evaluation of selected papaya genotypes for PRSV tolerance through artificial inoculation

To screen for PRSV resistance in diverse genotypes of papaya, thirty plants of each of the papaya genotypes such as Pune Selections (PS-1, PS -2, PS-3, and PS-5) and other commercial papaya cultivars (PM, Co2, Pusa Delicious, Pusa Dwarf, Madhu Bindu and Red Lady) and the papaya wild relative *Vasconcellea cauliflora* were planted in pots for artificial inoculation. To reconfirm the PRSV tolerance in the above listed genotypes of papaya, all the test lines were sap inoculated at 4–6 leaf stage using 0.1 M phosphate buffer, pH 7.5 with previously maintained PRSV isolate in a susceptible papaya cv Red Lady in the insect proof glasshouse at IARI Regional Station, Pune (Gorane et al., 2019). Sap inoculated plants were kept in insect proof glasshouse for 4–6 weeks for recording the symptoms and further confirmation and quantification of PRSV.

### Detection and quantification of PRSV by ELISA and RT-PCR

The leaf tissues from fully expanded young leaves of two papaya genotypes, PM (Susceptible) and PS 3 (tolerant) were collected from healthy and PRSV infected plants maintained at ICAR-IARI, Regional Station, Pune (Maharashtra, India). Leaves of the PRSV resistant wild relative of papaya *V. cauliflora*, were also collected for comparison. The presence and absence of PRSV infection in collected leaf samples was confirmed by ELISA and RT-PCR using PRSV specific antisera and primers, respectively. In order to confirm the virus infection, leaf samples from papaya plants were collected 4 weeks post inoculation and subjected to double antibody sandwich-enzyme linked immunosorbent assay (DAS-ELISA) using PRSV specific polyclonal antibodies (Agdia Inc., USA). Further reconfirmation of inoculated samples was done by reverse transcriptase polymerase chain reaction (RT-PCR) on selected samples of each papaya line. Total RNA was extracted from selected leaf samples by RNeasy plant mini kit (Qiagen, Germany) and cDNA synthesized using iScript™cDNA synthesis kit (Biorad, USA) following manufacturer’s instructions. The PRSV CP gene was amplified by PCR using 2 μl cDNA template, 12.5 μl of PCR master mix (ThermoFisher Scientific, USA) and 10pM of each PRSV CP specific primers (Gorane et al., 2021). PCR program was setup as follows: 1 cycle of initial denaturation for 3 min at 94°C followed by 35 cycles of denaturation at 94°C for 30 sec, annealing at 60°C for 1 min and extension at 72°C for 1 min and 1 cycle of final extension at 72°C for 10 min. The PCR products were subjected to electrophoresis in a 1% (w/v) agarose gel, stained with GelRed nucleic acid stain (Biotium, USA) and visualized in Gel Documentation system (Syngene, UK).

### RNA extraction, library construction and sequencing

Total RNA was extracted from the papaya leaves using Sigma Plant RNA isolation kit (Sigma-Aldrich, USA), according to the manufacturer’s instructions. The small RNA library was constructed by the Small RNA Sample Pre-kit. The small RNA samples were ligated by using chimeric oligonucleotides 5’-GTTCAGAGTTCTACAGTCCGACGATC-3’ and 3’-TCTGCACACGAGAAGGCTAGA-5’ with adapter sequences. The final cDNA library was ready for sequencing, after a round of adapter ligation, reverse transcription, PCR enrichment, purification and size selection. The qualified library was subsequently sent for Illumina HiSeq2500 sequencing to Nucleome Informatics Pvt. Ltd. (Hyderabad, India). The raw reads generated were processed by removing the adapter sequences, empty reads, no insert tags, oversized insertion, low quality reads (>50% of the bases with a quality score =5), poly A tags and small tags to obtain the clean reads (**Supplementary Figure 1**). The clean reads were further filtered based on read length of 18–24 nucleotides using in-house scripts.

### Identification and differential expression analysis of miRNAs

Clean reads were first aligned with PRSV P isolate DEL (Accession No. EF017707, Parameswari et al., 2007) in which unaligned reads were considered for further analysis. Unaligned reads were mapped to the *C. papaya* hairpin loop of miRBase v21.0 (https://mirbase.org/) to filter known miRNAs. Known miRNA count files and unaligned reads from the above process were submitted to miARma-seq (miRNA-Seq and RNA-Seq Multiprocess Analysis) suite to predict novel miRNAs. This suite was used to identify differential expression analysis of known and novel miRNAs. The miARma-Seq (Andrés-León et al., 2016) uses edgeR software for differential expression analysis which includes: Read counts Normalisation, Model dependent p-value estimation and FDR value estimation based on multiple hypothesis testing. The significant differentially expressed known and novel miRNAs were filtered based on FDR < 0.05.

### Target identification and functional annotation

The targets of some of the differentially expressed plant miRNAs were identified through the online tool psRNATarget (released 2017; https://www.zhaolab.org/psRNATarget/home) by using sequence of papaya (*C. papaya*) transcript, JGI genomic project, Phytozome, phytozome v8.0, internal number 113 (Dai et al., 2018). The psRNATarget server runs on a Linux cluster with a robust distributed computing back-end pipeline and is developed for high-throughput analysis of the NGS data. This software encompasses latest findings of plant miRNA target recognition, that can distinguish the translational and post-transcriptional inhibition, thus with an ability to report the number of small RNA/target site pairs that can affect small RNA binding activity to target transcript. The psRNATarget evaluates complementarity between small RNA and target gene transcript using the scoring scheme originally applied by miRU (Zhang, 2005). The targets with single base or no mismatches in the seed region (position 2–8 bases) were considered for further analysis. The functional identification of targets was performed using functional annotation tools agriGo (Gene Ontology Analysis Toolkit and Database for Agricultural Community) (Tian et al., 2017).

### Validation of small RNA data by RT-PCR for selected miRNAs

**Figure 1:**
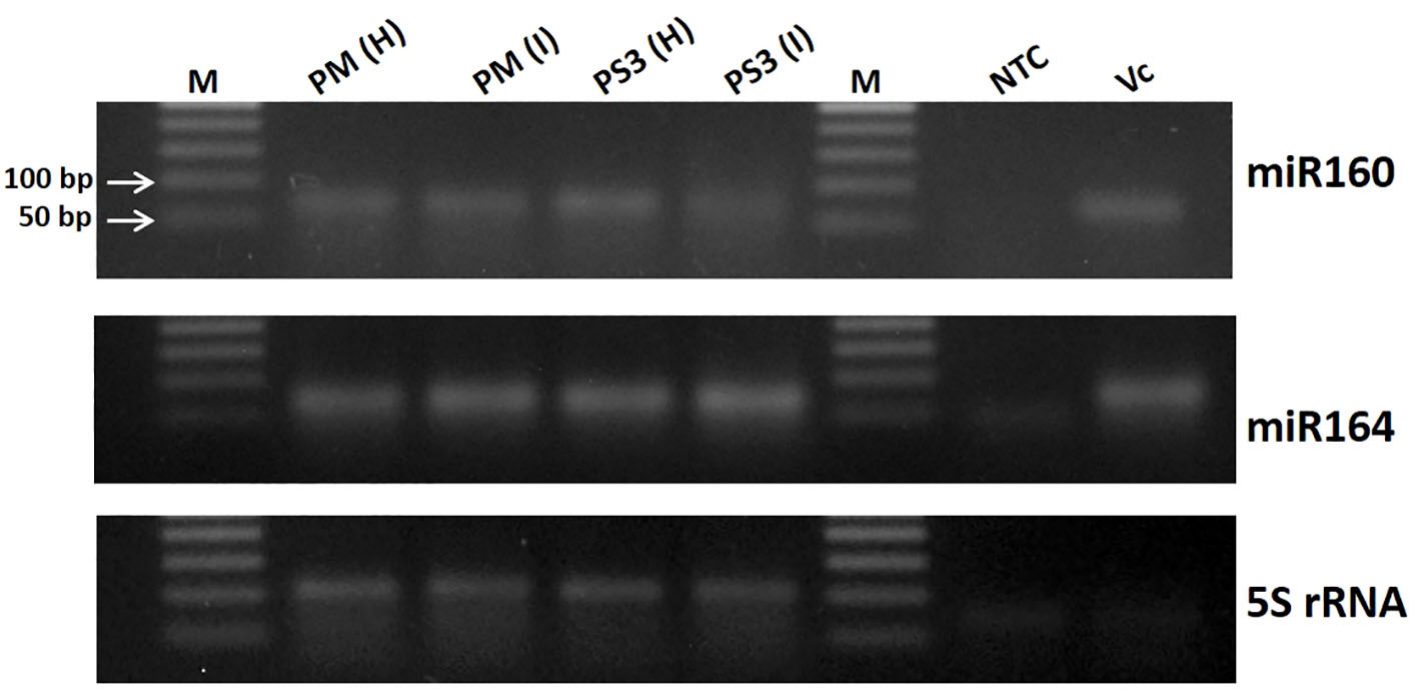
Validation of small RNA data obtained from different papaya genotype leaf samples by next generation sequencing using semi-quantitative RT-PCR. M, DNA ladder; PM-H, Pusa Majesty-Healthy; PM_I, PM-Infected; PS3_H, Pune Selection 3 – Healthy; PS3_I, Pune Selection 3 – Infected; NTC, Negative Control and VC, *Vasconcellea cauliflora*.

The small RNA data obtained from different papaya samples by next generation sequencing was validated using semi-quantitative RT-PCR (**Figure 1**). The total RNA was extracted from the papaya leaf samples using Spectrum Plant Total RNA Kit (Sigma-Aldrich, St. Louis, MO, USA) following the manufacturer’s instructions. About 2 µg of total RNA was subjected to reverse transcription using the MultiScribe Reverse Transcriptase, RT Random Primers and other components from the High-Capacity cDNA Reverse Transcription kit (Applied Biosystems, Foster City, CA, USA). The RT-PCR reactions were carried out in a total reaction volume of 20 µL, with a cDNA synthesis cycle of 25 °C for 10 min, 37 °C for 120 min and 85 °C for 5 min, set up in a Bio-Rad S1000 Thermal cycler (Bio-Rad Laboratories, Hercules, CA, USA). A 1:10 dilution of the cDNA was used for PCR amplification of various miRNAs (miR160, miR164 and 5S rRNA), using specific primer pairs (**Supplementary Table 1**). The RT-PCR products were resolved in 2% TAE (Tris-acetate-EDTA) agarose gels stained with ethidium bromide and visualized under ultraviolet (UV)-light.

## Results

### Evaluation of papaya lines for tolerance to papaya ringspot virus

**Figure 2:**
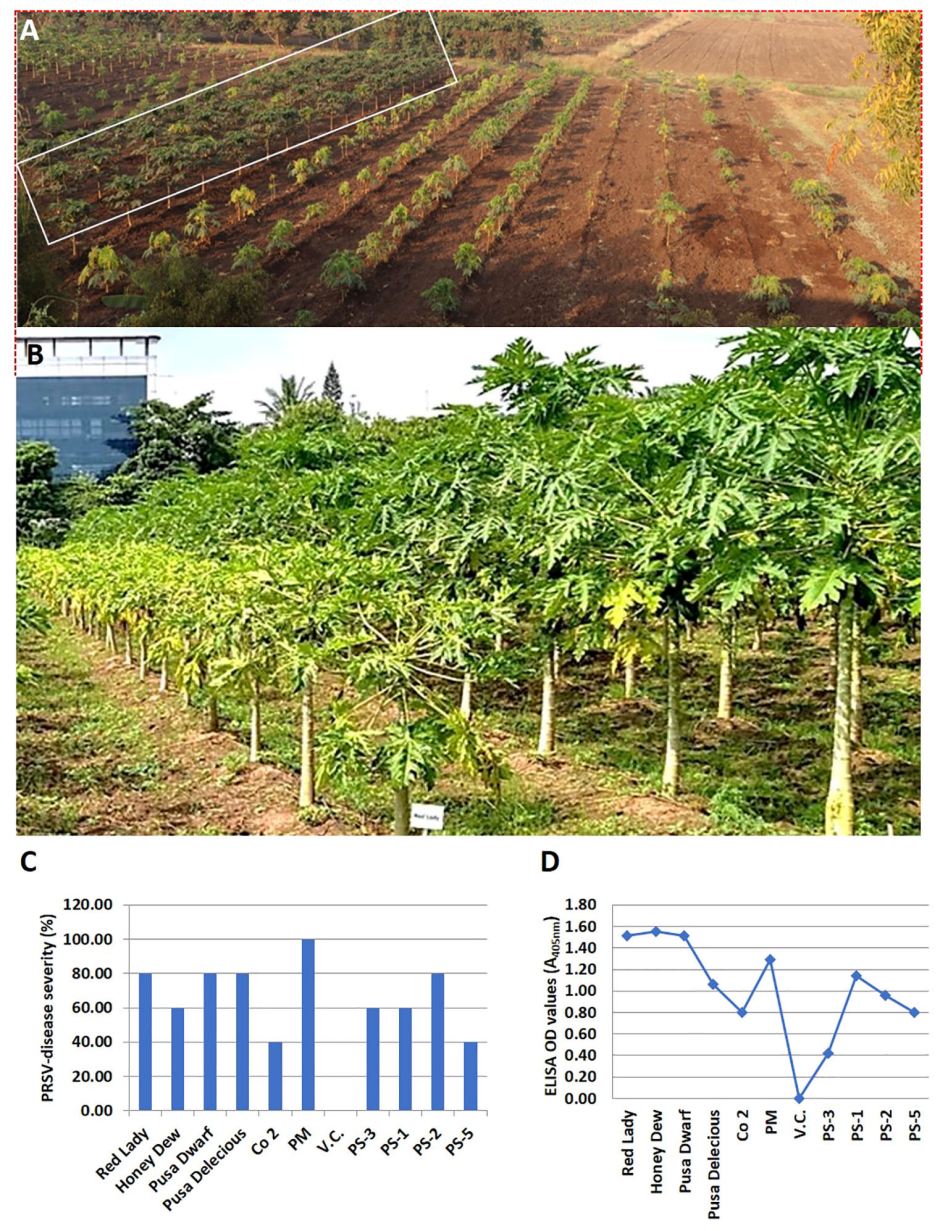
Performance of various papaya genotypes for PRSV resistance at the IARI experimental Station, Pune, Maharashtra state, India. **(A)** The papaya plant rows that are marked in a rectangular box are the PRSV tolerant Pune selection (PS) papaya lines, while the papaya plant rows on its right side are the PRSV susceptible commercial papaya varieties. **(B)** Performance of PRSV tolerant Pune selection line (right row) vs susceptible commercial variety (left row) at fruiting stage. **(C)** Levels of PRSV infectivity, in percentage, as exhibited by different papaya genotypes in glasshouse by artificial PRSV inoculation, **(D)** Graphical representation of PRSV load in artificially inoculated papaya genotypes under glasshouse condition, as determined by ELISA (OD value).

A total of 11 papaya genotypes (6 commercial cultivars, 4 Pune Selections (PS) and one wild relative of papaya *V. cauliflora*) were evaluated for resistance to PRSV, at IARI, Regional Station Pune (Maharashtra state, India). Overall, the PRSV-disease incidence recorded was highest (100%) in commercial cultivar PM and lowest (40%) in PS5, followed by PS3. The overall average PRSV disease incidence was higher (73%) in commercial cultivars as compared to PS lines where average disease incidence was 60%. The presence of PRSV infection was confirmed in all the test genotypes by both ELISA and RT PCR. However, the virus titer varied in different papaya lines. The highest ELISA OD value of 1.55 was recorded in Honey Dew and the lowest value of 0.42 in PS3 papaya line. These OD values varied from 0.8 to 1.55 in commercial papaya test lines. Overall, the average ELISA value was recorded higher (1.29) in commercial papaya cultivar as compared to PS lines (0.83). Moreover, the field experiments over the years for evaluation of PRSV resistance showed similar pattern of disease intensity as reported by Sharma and Tripathi (2019). The above finding along with previous reports of field experiments showed that the PS lines have a good level of tolerance against PRSV infection as compared to the other commercial papaya cultivars (**Table 1**; **Figure 2**).

**Table 1:** Field evaluation of different papaya genotypes for tolerance to Papaya ringspot virus (PRSV) infection and estimation of PRSV titer by ELISA and RT-PCR.

Papaya genotype/cultivar	PRSV infectivity in glasshouse (%)	PRSV titer estimation by ELISA (OD value)	PRSV confirmation by RT-PCR	Type of PRSV Symptoms observed
Red Lady	80	++ (1.51)	+	Mild Mosaic
Honey Dew	60	++(1.55)	+	Mosaic
Pusa Dwarf	80	+ +(1.51)	+	Mosaic
Pusa Delicious	80	+ (1.06)	+	Mosaic
Co-2	40	+ (0.8)	+	Mild mosaic
PM	100	+ (1.29)	+	Mosaic
V.C.	0	–	–	No Symptoms
PS-3	60	+ (0.42)	+	Mild mosaic
PS-1	60	+(1.14)	+	Mild mosaic
PS-2	80	+ (0.96)	+	Mild mosaic
PS-5	40	+ (0.80)	+	Mild mosaic

Pusa Majesty (PM), Pune Selections (PS)-1, 2, 3 & 5, and *Vasconcellea cauliflora* (VC). Higher virus titer (++), Moderate virus titer ( +), Virus not detected (-).

### Analysis of small RNA sequencing data

To study the impact of PRSV infection with reference to different miRNAs and transcriptomic changes at genome-scale, two different papaya varieties PM (PRSV susceptible) and PS3 (PRSV tolerant) were used along with *V. cauliflora* (VC; PRSV resistant). For further characterization, Illumina HiSeq2500 sequencing platform was used to sequence the healthy and infected PM and PS3, together with VC, which generated a total of 237.8 million reads. Comparable numbers of reads were obtained in healthy samples of PRSV susceptible (PM) and a tolerant (PS3) variety i.e. 52 million reads while VC and infected samples ranged from 42–45 million reads (**Table 2**).

**Table 2:** Summary of type and number of small RNA reads obtained by Illumina HiSeq 2500 sequencing in healthy and infected Papaya varieties PM (PRSV susceptible), PS3 (PRSV tolerant) and VC (Wild relative of papaya).

	Healthy Papaya Plants			PRSV infected Plants	
RNA class	PM (H)	PS3 (H)	VC	PM (I)	PS3 (I)
Raw reads	52,989,070	52,663,396	42,988,993	43,368,829	45,873,913
Total Clean reads	52,841,249	52,543,607	42,876,839	43,262,152	45,749,345
**Clean reads** (18–24 nt)	12,156,513	21,983,529	16,305,777	34,217,191	24,350,232
Unaligned reads	11,994,524	21,021,736	16,164,592	31,146,449	22,893,062
Novel miRNA reads	139,567	419,784	114,025	4,371,533	711,008
Known miRNA reads	160,874	943,881	139,427	1,145,855	688,181
Virus alignment	1,115	17,912	1,758	1,924,887	768,989

Pusa Majesty (PM), Pune Selections-3 (PS- 3), and *Vasconcellea cauliflora* (VC); H, healthy; I, PRSV infected.

A high percentage (99%) of clean reads were obtained after further processing, filtering and mapping of raw reads with the reference genome. These clean sequences were further classified as unaligned reads and virus aligned reads (**Table 2**). The unaligned sequences were varying in different samples, 71% clean reads were unaligned in PM(I), 50% in PS3(I), 40% in PS3(H), 37.7% in VC, and 22.6% in PM(H) (**Table 2**). A maximum of 4.3 million reads of novel miRNAs was obtained after mapping unique and unaligned reads for PM(I), whereas PS3(I) had only 0.711 million reads of novel RNAs (**Table 2**).

**Figure 3:**
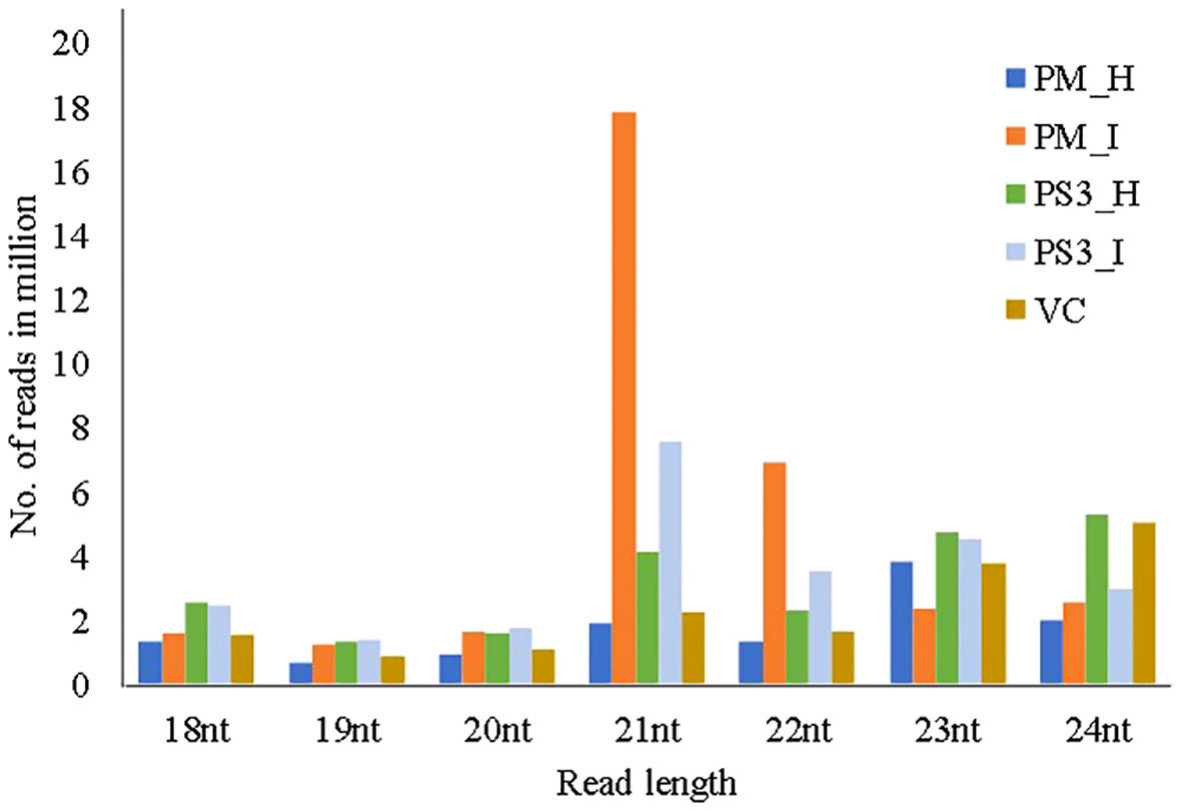
Size distribution of small RNAs, with read lengths in the range of 18–24nts, retrieved through Illumina next generation sequencing from different papaya genotypes that show different levels of tolerance to PRSV. PM-H, Pusa Majesty-Healthy; PM_I, PM-Infected; PS3_H, Pune Selection 3 – Healthy; PS3_I, Pune Selection 3 – Infected; and VC, *Vasconcellea cauliflora*.

The size distribution of the sequenced small RNA ranged from 18 to 24 nucleotides (nt) (**Figure 3**). The abundance of small RNA reads is not identical in all the samples, 21nt sequence was predominant in PRSV infected samples of PM and PS3, while 23nt was dominant in PM-H, 24nt were dominant in PS3-H and VC. Whereas the smaller sized small RNA species i.e., 18–20nt were in reduced amounts in all the plant samples irrespective of PRSV infection status (**Figure 3**).

### Identification and quantification of known and novel miRNAs

**Figure 4:**
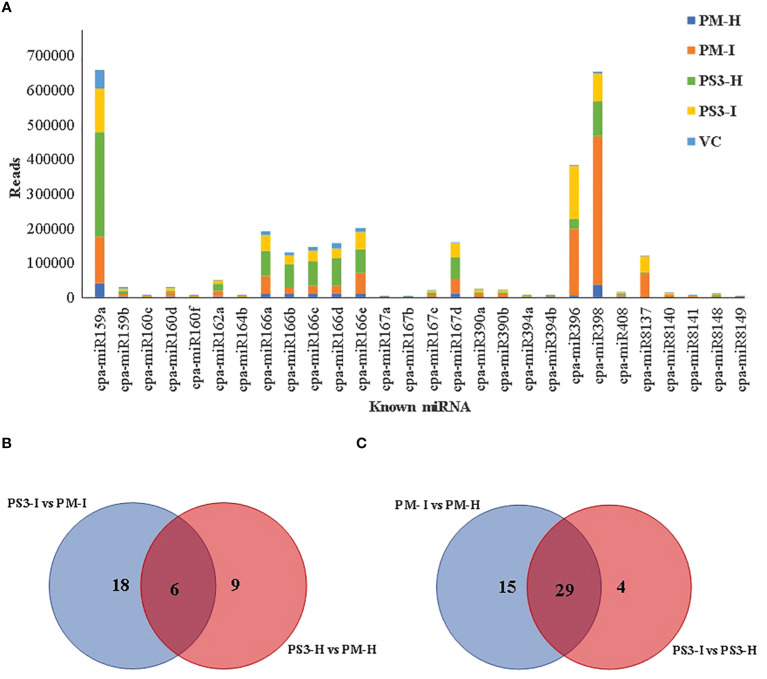
Differentially expressed miRNAs between PRSV infected and non-infected papaya genotypes: **(A)**. The known miRNAs showing more than 1000 reads in any one of the samples, **(B)** and **(C)** Venn diagrams showing known differentially expressed miRNAs in Healthy and infected, susceptible and resistant papaya cultivars. PM-H, Pusa Majesty-Healthy; PM_I, Pusa Majesty-Infected; PS3_H, Pune Selection-3 – Healthy; PS3_I, Pune Selection-3 – Infected; and VC, *Vasconcellea cauliflora*.

In order to filter known miRNAs, the unique sequences were further mapped to the *C. papaya* hairpin loop of miRBase v21 (https://mirbase.org/) and were quantified using in-house scripts. According to the alignment results, the maximum number of reads were noted in the PM (I) sample i.e. 1,145,855 while the healthy samples PM(H) showed 160,874 reads (**Table 2**; **Figure 4A**). The wild relative of papaya *V. cauliflora* showed the lowest reads of 114,025.

It was interesting to note that the PRSV tolerant papaya variety PS3 showed higher reads in healthy samples as compared to the PRSV infected samples (**Table 2**). A total of 79 known miRNAs were identified among different samples, 28 known miRNAs show more than 1000 reads (**Figure 4A**). Maximum number of reads were recorded for the two microRNAs, miR159a and miR398 (**Figure 4A**; **Table 3**). A comparison between PRSV resistant and susceptible papaya varieties suggests that the 18 miRNAs were differentially expressed in infected PS3 and PM samples, while 9 miRNAs were differentially expressed in healthy samples and 6 miRNAs were present in both healthy as well as the infected samples (**Figure 4C**). Further, the infected and healthy samples were compared with each other, wherein 15 known miRNAs were identified in PM variety that were not present in PS3, while 4 miRNAs were differentially expressed in PS3 alone (**Figure 4B**). On the other hand, 29 known miRNAs were differentially expressed in both the varieties (**Figure 4C**). The known miRNA count files and unaligned reads from the above process were submitted to miARma-seq suite to predict novel miRNAs (mirDeep of MiARma-Seq suite). A total of 291 novel miRNAs were identified in different papaya genotypes (**Table 4**).

**Table 3:** Differential expression of known miRNAs in healthy and PRSV infected Papaya plant leaf samples and their comparative expression is denoted as ↑ (boxed in green color) as upregulation, ↓ (boxed in red color) as downregulation.

	PM- I vs PM-H	PS3-I vs PS3-H	PS3-H vs PM-H	PS3-I vs PM-I	PM-I vs VC	PS3-I vs VC	VC vs PM-H	VC vs PS3-H
cpa-miR156a	↑	↑	−	↓	↑	↑	↓	↓
cpa-miR156b	↑	↑	−	↓	↑	↑	↓	↓
cpa-miR156c	↑	↑	−		↑	↑	↓	↓
cpa-miR156e					↓	↓	↑	↑
cpa-miR156f					↓	↓	↑	↑
cpa-miR159a	↓	↓	−		↓	↓	↑	
cpa-miR159b	↓	−	−		↓	↓	↑	↑
cpa-miR160a	−	↑	↓	↑	↓	↓	↑	↑
cpa-miR160b	−	↑	↓	↑	↓	↓	↑	↑
cpa-miR160c	↑	↑	↓		↑		↑	↑
cpa-miR160d	−	↑	↓		↑	↑	↓	↓
cpa-miR160e	↓	↑	↓	↑	↓	↓	↑	
cpa-miR160f	↑	↑	↓		↑	↑	↑	↑
cpa-miR162a	↑	−	↑				↑	
cpa-miR164a	−	↑	↓		↑	↑		
cpa-miR164b	↑	↑	−		↑	↑	↓	
cpa-miR164c	−	↑	↓		↑	↑	↓	
cpa-miR164d	−	↑	−			↑		
cpa-miR164e		↑			↑	↑		
cpa-miR166b	↓	↓	−		↓	↓		
cpa-miR166c	↓	↓	−	↑	↓	↓		
cpa-miR166d	↓	↓	−	↑	↓	↓	↑	↑
cpa-miR167a	↓	−	−					
cpa-miR167b	↓	−	−					
cpa-miR167c	↓	↓	−		↓			
cpa-miR167d	↓	−	−		↑	↑	↓	↓
cpa-miR169					↓	↓	↑	↑
cpa-miR171a					↓	↓	↑	↑
cpa-miR171b	↓	−	−	↑	↓	↓	↑	↑
cpa-miR171c				↑	↓	↓	↑	↑
cpa-miR171d	↑	↑	−		↓	↓	↑	↑
cpa-miR172a					↓			
cpa-miR172b					↓	↓	↑	↑
cpa-miR319	↑	−	−	↓	↓	↓	↑	↑
cpa-miR390a	↑	↑	−			↑		
cpa-miR390b	↑	↑	−		↑	↑	↑	↑
cpa-miR393	↑	↑	−		↑	↑		
cpa-miR394a	↓	↓	−		↓	↓		
cpa-miR394b	↓	−	−					↑
cpa-miR395a	↑	↑	−		↓	↓	↑	↑
cpa-miR395b	↑	↑	−		↓		↑	
cpa-miR395c		↑			↓	↓	↑	↑
cpa-miR395d	↑	↑	−	↓	↓	↓	↑	↑
cpa-miR395e	↑	↑	−	↓	↓	↓	↑	↑
cpa-miR396	↑	↑	−		↑	↑	↓	↓
cpa-miR398	↑	−	↓	↓	↑	↑	↓	↓
cpa-miR408	↓	↓	↓	↓	↑	↑	↓	↓
cpa-miR477	↓	↑	↓	↑	↑	↑	↓	↓
cpa-miR5211	↑	−	−		↑			
cpa-miR535	↑	↑	−					
cpa-miR8134	−	↑						
cpa-miR8135	↑	↑	−	↓	↑	↑	↓	↓
cpa-miR8136					↑	↑	↓	↓
cpa-miR8137	↑	↑	↓		↑		↓	↓
cpa-miR8139a				↓	↑	↑	↓	↓
cpa-miR8139b		↑				↑	↓	
cpa-miR8139c					↑			
cpa-miR8139d					↑	↑	↓	↓
cpa-miR8139e				↓	↑	↑	↓	↓
cpa-miR8140	↑	↑	−		↓	↓	↑	↑
cpa-miR8141					↑	↑	↓	↓
cpa-miR8142					↑	↑	↓	↓
cpa-miR8143		↑				↑		
cpa-miR8144	↓	↓	−	↑	↑	↑	↓	↓
cpa-miR8145					↑	↑	↓	↓
cpa-miR8146				↓	↑	↑	↓	↓
cpa-miR8148	↓	↓	−	↑	↑	↑	↓	↓
cpa-miR8149	↓	−	−		↑	↑	↓	↓
cpa-miR8150		↓			↑	↑	↓	↓
cpa-miR8151					↑	↑	↓	↓
cpa-miR8152	↓	−	↓		↑	↑	↓	↓
cpa-miR8153	↓	−			↑	↑	↓	↓
cpa-miR8154	↑	↑		↓	↑	↑		
cpa-miR8155	−	↓	↑		↓	↓	↑	

Pusa Majesty (PM), Pune Selection-3 (PS3), and Vasconcellea cauliflora (VC).

**Table 4:** Differential expression of known and novel miRNAs in healthy and infected papaya varieties: Pusa Majesty (PM), Pune Selection-3 (PS3), and *Vasconcellea cauliflora* (VC).

	Known miRNAs			Novel miRNAs		
Samples	Up	Down	Total	Up	Down	Total
PM-I vs PM-H	24	20	44	37	15	52
PM-I vs PS3-H	36	17	53	35	14	49
PM-I vs VC	38	27	65	40	8	48
PS3-I vs PM-H	18	15	33	40	6	46
PS3-I vs PS3-H	33	11	44	34	9	43
PS3-I vs VC	39	25	64	40	7	47
VC vs PM-H	26	29	55	7	14	21
VC vs PS3-H	23	27	50	4	17	21

### Differential expression of miRNAs associated with PRSV infection

A comparative analysis of change in the levels of expression of miRNAs in the infected samples vs healthy samples indicated that the higher number of miRNAs were upregulated in infected samples. A similar pattern was noticed in both known as well as novel miRNAs (**Table 4**).

#### Known miRNAs

**Figure 5:**
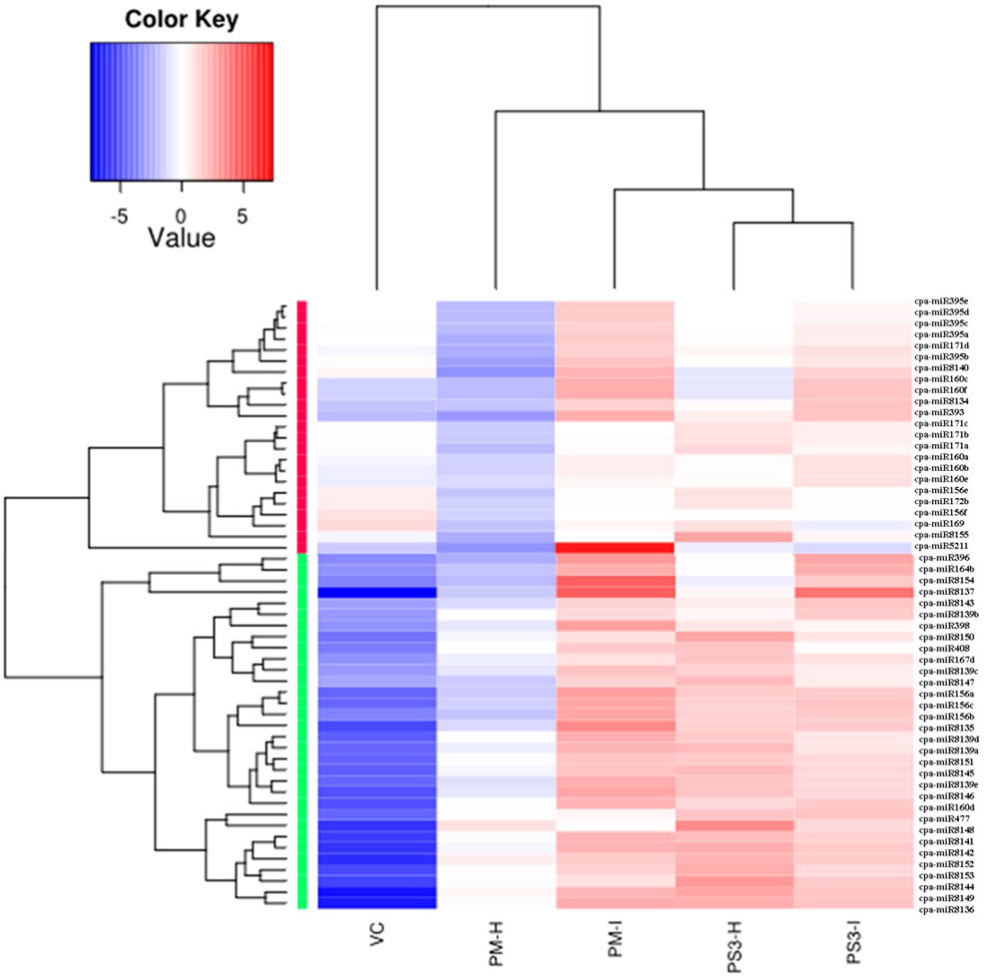
Heat map obtained from sequencing data showing differentially expressed known miRNAs in PRSV infected leaves (PM-I and PS3-I) to healthy samples (PM-H, PS3-H) and VC. cpa-miR stands for microRNA of *Carica papaya* plant. The color scale shown at the top illustrates relative expression of a miRNA across all samples: red represents expression above mean, blue represents expression lower than mean. PM-H, Pusa Majesty-Healthy; PM_I, PM-Infected; PS3_H, Pune Selection 3 – Healthy; PS3_I, Pune Selection 3 – Infected; and VC, *Vasconcellea cauliflora*.

A total of 44 miRNAs were compared of which 24 were upregulated and 20 were downregulated in the PRSV infected susceptible papaya variety PM. The heat map of the data clearly suggests that the conserved miRNAs belonging to family miR395, miR171, miR160, miR156, miR172, miR169, miR164, miR396, miR398, miR408, miR167 and miR477 show differential expression in infected and healthy samples (**Figure 5**). Amongst the conserved miRNAs, miR396, miR160f, miR160c, miR390b, miR390a, miR393, miR398, miR164b, miR156a, miR156b, miR156c, miR171d, miR395b, miR395a, miR395d, miR395e, miR162a, miR319, miR535 were upregulated, whereas miR477, miR166c, miR166d, miR166b, miR408, miR159a, miR167d, miR167c, miR167b, miR167a, miR160e, miR171b, miR159b and miR394b were downregulated in PRSV infected PM samples as compared to PM-H. Some of the miRNA specifically present in papaya such as miR8137, miR8140, miR8154, miR8135 are upregulated while miR8148, miR8152, miR8153, miR8149 and miR8144 were downregulated in infected samples as compared to healthy samples of PM (**Figure 5**).

The PRSV tolerant variety (PS3) shows contrasting expression as compared to susceptible papaya variety (PM) upon PRSV infection. The miRNAs miR408, miR166b, miR166c, miR166d, miR159a, miR477, miR167d, miR167c, miR8148, miR8152, miR8149, miR8153 are upregulated in PS3-I, whereas they get downregulated in PM-I when compared to their corresponding healthy samples (**Table 3**). Similarly, contrasting expression pattern was noted for the miRNAs: miR396, miR160f, miR160c, miR390b, miR390a, miR393, miR164b, miR156a, miR156b, miR156c, miR171d, miR395b, miR535, miR8137, miR8140, which gets downregulated in infected samples of PS3, while upregulated in PM-I. The miR398 is upregulated in infected samples of both the papaya varieties (**Table 3**).

Apart from contrasting results the miRNA identified only in PS3 variety includes miR160d which is upregulated, and miR164a, c, d that is downregulated in PRSV infected papaya samples (**Table 3**). A comparison between healthy samples of the papaya varieties PM and PS3 suggests differential expression of 15 miRNAs, among them the 2 miRNAs, miR162a and miR8155 were upregulated while 13 were downregulated (**Table 3**). The PRSV infected sample PS3 when compared with PM-I showed higher expression of 10 miRNAs while lower expression of 14 miRNAs. The miR160 a,b and miR171c were present only in the infected samples of PS3 and not in PM-I (**Table 3**).

Another comparison of miRNA expression was made between the PM, PS3 with the wild relative of papaya VC (PRSV resistant). Interestingly both the papaya varieties PM and PS3 showed similar miRNA expression pattern (**Table 3**). The expression of miR156 a,b,c, miR160c,d, miR160f, miR164a-e, miR167d, miR390a,b, miR393, miR396, miR398, miR408, miR477, miR5211, miR8135–37, miR8139a-e, miR8141–46, miR8148–54, were downregulated in healthy samples of both the papaya varieties. Some of the miRNAs which were downregulated in infected samples when compared to VC include miR156e-f, miR159a-b, miR160a,b, miR160e, miR166b-d, miR167c, miR169, miR171a-d, miR172a-b, miR319, miR394a, miR395a-e, miR8140, and miR8155 (**Table 3**).

#### Novel miRNAs

A total of 291 novel miRNAs were identified in the infected and healthy papaya leaf samples of contrasting genotypes and the wild relative. The sequencing reads suggest differential expression of 52 and 43 novel miRNAs in PM and PS3 respectively. Amongst these 29 miRNAs were upregulated and 8 miRNAs were downregulated in infected samples of both the papaya varieties, while 5 miRNAs showed differential expression. Two miRNAs unique for the papaya variety PM-I were downregulated, while one unique miRNA was upregulated in PS3-I samples. The PRSV resistant *V. cauliflora* samples were also sequenced and a comparison was made with the healthy samples of both the papaya varieties (**Table 3**). Twenty-one miRNAs were differentially expressed, 10 miRNAs showed similar expression, 2 miRNAs were upregulated, and 8 miRNAs were downregulated in VC when compared with PM and PS3 varieties (**Table 3**).

#### Target analysis of miRNAs

The miRNAs are negative regulators of gene expression, therefore, to investigate the role of miRNA, it is important to predict the target transcript sequences. The conserved miRNAs are associated with conserved targets that are common in various plant species such as Arabidopsis, rice, and poplar (Liang et al., 2013). The subfamilies of miRNAs differing at 1–2 nucleotide positions target same transcript, miR160a-f targets auxin response factors (ARF10, 16, 17), miR156a-c targets Squamosa promoter-binding-like protein 7,2, and the miR166b-d targets homeobox leucine zipper protein. Whereas the miR164a-c targets NAC domain containing protein, and the miR171b-d targets Scarecrow-like protein, histone lysine N-methyltransferase ATX2-like protein. The miRNAs are downregulated in VC as compared with papaya, that targets transcription factors of family SPL, ARF, NAC domain, growth factors (**Supplementary Table 2**). These transcription factors are involved in leaf development and lateral root development. The CpARF10, CpARF16, and CpARF17 showed fruit-specific expression, which indicated that they might play an important role in fruit ripening (Liu et al., 2015a).

## Discussion

MicroRNAs (miRNAs), are long noncoding RNAs, that regulate gene expression by binding to the target mRNAs, resulting in mRNA cleavage or inhibition of protein translation (Ambros, 2001; Carrington and Ambros, 2003; Bartel, 2004). The plant miRNAs play a crucial role in diverse biological phenomenon, such as development of leaf (Rhoades et al., 2002; Chen, 2005; Guo et al., 2005; Lauter et al., 2005), nutrient homeostasis (Chiou, 2007) and various stress responses (Chapman et al., 2004; Jones-Rhoades and Bartel, 2004). Understanding the differential expression pattern of miRNAs and their role during the virus infection in papaya will provide the clues to design advanced strategies to control the PRSV infection in papaya and other high value horticultural crops.

Therefore, in this study, we present a detailed analysis of the miRNA expression pattern in papaya plants that are susceptible (PM) and tolerant (PS3) to PRSV, with or without PRSV infection/symptoms, along with the wild relative of papaya *V. cauliflora*, to understand the miRNA-mediated PRSV resistance. We performed deep sequencing by employing Illumina HiSeq 2500 sequencing and compared the miRNA expression between the resistant and susceptible papaya genotypes, following PRSV infection. Here we also report and reconfirm similar findings of PRSV tolerance in papaya lines that was previously demonstrated by Sharma and Tripathi (2019), wherein the Pune Selection (PS) lines showed significant level of PRSV tolerance compared to the commercially cultivated papaya cultivar (PM: Pusa Majesty) under the field trials conducted for more than four years, at Pune in Maharashtra state (India). The present study is based on the past reports of Chavan et al. (2010), wherein eight commercial papaya cultivars were screened for PRSV resistance and a lowest PRSV incidence of 13.2%, was recorded for the papaya variety ‘Madhubala’, that subsequently served as parental material for the PRSV tolerant Pune Selections (PS). Both the present and the past studies reported a better tolerance to PRSV in PS lines when evaluated in different climatic conditions, at different time points. Thus, these PS lines can be a potential source of PRSV tolerance and can be incorporated in the future PRSV resistance breeding programmes. Further, *V. cauliflora*, that are wild relatives of cultivated papaya, were found to be immune to PRSV infection, which could be because of its natural immunity or non-host nature. As expected, PRSV was not detected in virus inoculated *V. cauliflora* plants, either by ELISA or by RT-PCR, which is in conformity with the previous reports (Sharma and Tripathi, 2019; Sharma et al., 2019). Based on the PRSV infection data from present and previously reported studies, PS3 can be a good candidate to further investigate the molecular basis of virus tolerance. Therefore, the two contrasting genotypes with reference to PRSV tolerance, PS3 and PM were selected to study the differential expression of known and novel miRNAs in response to PRSV infection, by employing next generation sequencing technologies. Some of the miRNA targets found in papaya are involved in the ubiquitin-dependent protein catabolic process and the increasing evidence indicates that plants utilize this process during their immune response to pathogen invasion. However, this ubiquitin pathway can also be used by the viruses to enhance the infection process, by enhancing their own replication (Marino et al., 2012). Infection of *N. tabacum* by the RNA viruses Tobacco etch virus (TEV) and Potato virus Y(PVY) representative of the *Potyviridae* family show higher expression of miR166, miR171, miR159, and miR167 post infection; while differential expression of miR160, miR169, miR164 and miR156 in TEV and PVY infected plants (Bazzini et al., 2007). Similarly, studies on another member of *Potyviridae* revealed that miR160, miR393, and miR1510 were involved in resistance to SMV infection in soybean plants (Yin et al., 2013). The small RNA (sRNA)-sequencing, degradome-sequencing, as well as a genome-wide transcriptome analysis in SMV infected soybean revealed that increase in the expression level of miR168 leads to a serious inhibition of the target AGO1 mRNA, that encodes for a member of the argonaute family of proteins, which associate with the small RNAs and have important roles in RNA interference (Chen et al., 2015, 2016). In another study, microarray analysis indicated that the up-regulated miRNAs, namely, miR168a, miR403a, miR162b and miR1515a regulated the expression of AGO1, AGO2, DCL1 and DCL2, that encode for the components of silencing complex (Bao et al., 2018). Studies by Abreu et al. (2014) on response of papaya microRNAs to infection by papaya meleira virus (PMeV) resulted in identification of 462 microRNAs, representing 72 microRNA families. The expression of 11 microRNAs, with potential targets in 20S and 26S proteasomal degradation pathway was studied in response to PMeV infection and its titer.

In the present study, the abundant reads of small RNA (23nt and 24nt) were predominant in PM-H, PS3-H and *V. cauliflora*. Whereas, the 21nt small RNA reads were predominant in PRSV infected leaf samples of PM and PS3. These results are similar to earlier reports which have shown that 24nt small RNAs are more abundant in several other plant-pathogen interactions such as tomato-late blight, tomato-cucumber mosaic virus (CMV) and wheat-powdery mildew pathogen (Xin et al., 2010; Feng et al., 2014; Luan et al., 2015). The distribution of different sized distinct small RNAs may reflect their compositions (Ding and Lu, 2011). In this study, we found that both 23nt and 24nt small RNAs were more abundant in the healthy papaya plants than in the PRSV infected papaya. Whereas the 21nt and 22nt sized miRNAs were more abundant in both the papaya varieties, PM and PS3 infected with PRSV. This finding is in line with reports by Du et al. (2019) where they found larger number of 24nt sized small RNAs in the non-infected library than in the virus infected. The expression profile of miRNAs was altered on co-infection of tobacco curly shoot virus along with its betasatellite in *N. benthamiana* (Du et al., 2019). A number of siRNAs and their targets have been shown to determine leaf development, such as miR156-Squamosa Promoter Binding Protein-Like (SPL) (Xu et al., 2016), miR160-Auxin Response Factor (ARF) (Ben-Gera et al., 2016), miR165/166-Class III Homeodomain-Leucine Zipper (HD-ZIPIII) (Jia et al., 2015), miR319-Teosinte Branched/Cycloidea/Proliferating Cell Factor (TCP) (Bresso et al., 2018), miR390-Trans-Acting Small Interfering RNA3 (TAS3) (Husbands et al., 2016) and miR396-Growth Regulating Factor (GRF) (Omidbakhshfard et al., 2015). Additionally, multiple crucial components that are required for miRNA and ta-siRNA biogenesis, such as AGO1 (Bohmert et al., 1998), AGO7 (Garcia et al., 2006), SERRATE (SE) (Prigge and Wagner, 2001), and Hyponastic Leaves 1 (HYL1) (Liu et al., 2011) are suggested to influence the leaf development. The miRNA expression levels are associated with the different types of leaf curvatures (Williams et al., 2005). The qPCR of miRNAs revealed that a higher level of miR166 was associated with the downward curvature of leaf, while higher miR319a expression was correlated with wavy margins of the leaves (Williams et al., 2005).

In our study, a total of 44 known miRNAs and 291 potentially novel miRNAs were found to be differentially regulated during PRSV infection in papaya. Out of the 44 known miRNAs 20 miRNAs were downregulated and 24 were upregulated. Several studies report that the miRNAs also regulate host defenses against pathogens, including viruses, by suppressing pathogen multiplication at the post-transcriptional level (Al Abdallat et al., 2014; Feng et al., 2014; Du et al., 2019). Several miRNAs (e.g., miR168, miR169, and miR482) have been reported to target transcription factors controlling host resistance to virus infection (Várallyay et al., 2010; Zhu et al., 2013; Li et al., 2017). In *N. benthamiana*, virus infection may regulate the expression of miR168 to alleviate the anti-viral function of AGO1 protein (Várallyay et al., 2010; Du et al., 2019). Our study also showed the differential expression of 15 miRNAs which might play a key role in resisting the PRSV infection, by manipulating some of the genes involved in metabolic or gene silencing pathway. Past studies had shown that the rice miR164 played an important role in resistance to southern rice black-streaked dwarf virus infection, as well as resistance to drought stresses by differentially regulating its target genes in rice crop (Feng et al., 2014; Xu et al., 2014). Du et al. (2019) had also shown the expression of miR164a and miR482 in the two libraries derived from *N. benthamiana* coinfected with TbCSV and TbCSB, suggesting that these two miRNAs may have an important role in virus resistance. Further, studies by Xiao et al. (2014) indicated for a role of miRNA-mediated production of phasiRNAs in interaction between begomovirus and the model host plant *N. benthamiana*. Various studies have demonstrated a role of miRNA-mediated phasiRNA pathway in diverse biological processes, including pathogen resistance (Kurihara et al., 2006; Kang et al., 2012; Peng et al., 2014; Cui et al., 2017; Zheng et al., 2020). The miR482/2118-mediated cleavage of NBS-LRR transcripts involved in disease resistance play an important role in non-race specific disease resistance and also triggers production of phased small RNAs that regulate the expression of their target genes (Kurihara et al., 2006; Kang et al., 2012). On infection by the virus, the expression level of miR482 was down-regulated and as a result the NBS-LRR transcripts were up-regulated (Kang et al., 2012; Cui et al., 2017). Analyzed 18 miRNA libraries prepared from SMV resistant and susceptible near-isogenic lines (NILs) of soybean, at three different time intervals of post virus infection. Their study found that a large number of miRNAs were differentially expressed in the two soybean NILs, that targeted a series of NBS-LRR resistance (R) genes. In a similar study by Soltani et al. (2021), the effect of CMV infection on different quinoa varieties, resulted in identification of differentially expressed miRNAs and the corresponding genes that modulated the variety-specific biological pathways, such as plant-pathogen interaction (PPI), DNA replication, repair and recombination, and hormone signaling. These biological pathways ultimately result in modulation of defense hormones such as SA, JA, and ET (Soltani et al., 2020). Some of the biological phenomenon, such as DNA replication and repair in the host genome are critical for antiviral mechanism (Soltani et al., 2020). Upregulation of DNA recombination resulted in persistent induction of LRR gene expression in Tobacco mosaic virus (TMV) infected tobacco plants ultimately leading to high level of resistance against TMV (Kovalchuk et al., 2003; Boyko et al., 2007).

Several studies have shown that the plant miRNAs target and negatively regulate the plant R genes by prompting the production of phased, trans-acting siRNAs (tasiRNAs) against these R genes, and this miRNA-mediated gene regulation is suppressed on bacterial or viral infection (Zhai et al., 2011; Li et al., 2012). In tobacco, the R gene “N” against TMV was found to be regulated by miR482 (Whitham et al., 1994; Li et al., 2012). In brief, the silencing of NBS-LRR genes by miR482, and their activation after miR482 down-regulation upon bacterial or viral infection, have been widely studied in different host plants (Li et al., 2012; Shivaprasad et al., 2012; Zhu et al., 2013). Similarly, Li et al. (2012) demonstrated that the expression of miR6019 and miR6020 in tobacco plants result in specific cleavage of transcripts of the N gene and its homologs by binding to the complementary sequence of the conserved Toll and Interleukin-1 receptors (TIR)-encoding domain of the N transcript (Li et al., 2012; Moon and Park, 2016). Moreover, synthesis of phased, secondary siRNAs (phasiRNAs) from the N coding sequence through overexpression of miR6019 was shown to be accompanied by reduction in the N gene transcript accumulation and N-mediated resistance against TMV (Li et al., 2012).

In present study, the expression of miRNAs was potentially regulated by PRSV infection in papaya plant lines that were characterized by using high-throughput sequencing technology. The molecular functions and validation of these miRNAs in inducing resistance against PRSV infection in the resistant plants requires further investigation. However, the results presented here will improve the understanding of the viral infection and the response of miRNAs in the host plants in general and in particular for PRSV in papaya plants. In the future, this knowledge will also be helpful in providing additional information for developing viral management strategies, mainly through the plant breeding technologies and biotechnological interventions.

## Data availability statement

The original contributions presented in the study are publicly available. This data can be found here: NCBI SRA, PRJNA1122089.

## Author contributions

BP: Conceptualization, Data curation, Formal analysis, Funding acquisition, Investigation, Methodology, Project administration, Resources, Software, Supervision, Validation, Visualization, Writing – original draft, Writing – review & editing. ST: Resources, Writing – review & editing.

## Supplementary material

Supplementary Figure 1 Workflow for the Illumina (HiSeq 2500) next generation sequencing data analysis.

Supplementary Table 1 Primers used for screening for PRSV and in the validation of NGS data by RT-PCR.

Supplementary Table 2 List of miRNAs upregulated/downregulated and the corresponding genes downregulated/upregulated along with the description of the putative target genes in Papaya.
