# Supplementary figures and images for: Differential expression of microRNAs in response to Papaya ringspot virus infection in differentially responding genotypes of papaya (Carica papaya L.) and its wild relative
Source: Front Plant Sci. 2024 Jun 20;15:1398437. doi: 10.3389/fpls.2024.1398437 (PMC11222417; doi:10.3389/fpls.2024.1398437)

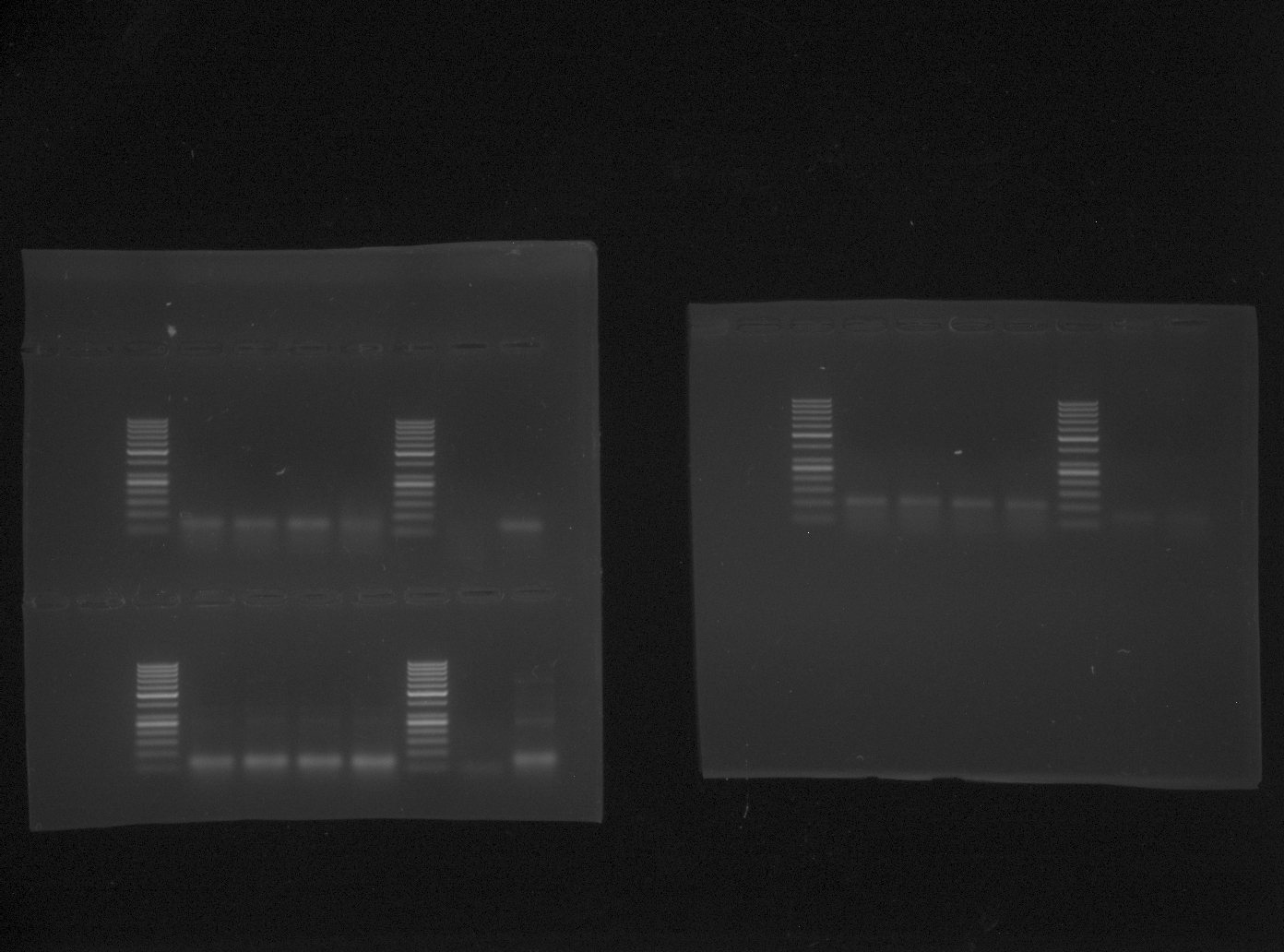

Supplement: Supplementary Figure 1 — Workflow for the Illumina (HiSeq 2500) next generation sequencing data analysis. [file Image_1.jpeg]
