# Supplementary material for: Differential expression of microRNAs in response to Papaya ringspot virus infection in differentially responding genotypes of papaya (Carica papaya L.) and its wild relative
Source: Front Plant Sci. 2024 Jun 20;15:1398437. doi: 10.3389/fpls.2024.1398437 (PMC11222417; doi:10.3389/fpls.2024.1398437)
Supplement: Supplementary Table 1 — Primers used for screening for PRSV and in the validation of NGS data by RT-PCR. [file Table_1.docx]

**Suppl. Table 1.** Primers used for screening for PRSV and in the validation of NGS data by RT-PCR.

| **Target Gene/miRNA** | **Primer Name** | **Primer Sequence (5’ to 3’)** | **Number of bases in the Primer** |
| --- | --- | --- | --- |
| PRSV-CP | CP-For | TCCAAAACTGAAGCTGTAGATGC | 23 |
|  | CP-Rev | GTTGCGCATACCCAGGAC | 18 |
| miR160 | Cpa-miR160c-RT | GTCGTATCCAGTGCAGGGTCCGAGGTATTCGCACTGGATACGACTGGCAT | 50 |
|  | Cpa-miR160c-For | GCGGCGGTGCCTGGCTCCCTGT | 22 |
| miR164 | Cpa-miR164b-RT | GTCGTATCCAGTGCAGGGTCCGAGGTATTCGCACTGGATACGACTGCACG | 50 |
|  | Cpa-miR164b-For | GCGGCGGTGGAGAAGCAGGGCA | 22 |
| 5S rRNA | Cpa-5SrRNA-RT | GTCGTATCCAGTGCAGGGTCCGAGGTATTCGCACTGGATACGACTTGATG | 50 |
|  | Cpa-5SrRNA-For | GTGCTTGGGTGAGATTAGTACTC | 23 |
