# Supplementary material for: Differential expression of microRNAs in response to Papaya ringspot virus infection in differentially responding genotypes of papaya (Carica papaya L.) and its wild relative
Source: Front Plant Sci. 2024 Jun 20;15:1398437. doi: 10.3389/fpls.2024.1398437 (PMC11222417; doi:10.3389/fpls.2024.1398437)
Supplement: Supplementary Table 2 — List of miRNAs upregulated/downregulated and the corresponding genes downregulated/upregulated along with the description of the putative target genes in Papaya. [file Table_2.docx]

**Suppl. Table 2.** List of miRNAs upregulated/downregulated and the corresponding genes downregulated/upregulated along with the description of the putative target genes in Papaya.

| **Sr. No.** | **miRNA upregulated** | **Gene Targets downregulated** | **Description of the Gene** |
| --- | --- | --- | --- |
| 1 | cpa-miR8153 | evm.model.supercontig_19.119\|pacid:16411849 | laccase-12 [*Carica papaya*] |
| 2 | cpa-miR166b/c/d | evm.model.supercontig_109.22\|pacid:16405536 | homeobox-leucine zipper protein ATHB-15 [*Carica papaya*] |
| 3 | cpa-miR166b/c/d | evm.model.supercontig_545.3\|pacid:16422471 | homeobox-leucine zipper protein REVOLUTA [*Carica papaya*] |
| 4 | cpa-miR394a | evm.model.supercontig_43.69\|pacid:16420029 | F-box only protein 6 [*Carica papaya*] |
| 5 | cpa-miR159a | evm.model.supercontig_131.73\|pacid:16408071 | uncharacterized protein LOC110808943 isoform X1 [*Carica papaya*] |
| 6 | cpa-miR159a | evm.model.supercontig_12.292\|pacid:16406850 | transcription factor MYB120-like [*Carica papaya*] |
| 7 | cpa-miR159a | evm.model.supercontig_34.210\|pacid:16417950 | transcription factor GAMYB-like [*Carica papaya*] |
| 8 | cpa-miR159a | evm.model.supercontig_33.151\|pacid:16417599 | transcription factor MYB33 [*Carica papaya*] |
| 9 | cpa-miR159a | evm.model.supercontig_12.16\|pacid:16406703 | protein SPOROCYTELESS-like [*Carica papaya*] |
| 10 | cpa-miR159a | evm.model.supercontig_7.22\|pacid:16425131 | SPOROCYTELESS-like EAR-containing protein 4 [*Carica papaya*] |
| 11 | cpa-miR160d | evm.model.supercontig_53.88\|pacid:16422316 | auxin response factor 16-like [*Carica papaya*] |
| 12 | cpa-miR160d | evm.model.supercontig_65.4\|pacid:16424448 | LOW QUALITY PROTEIN: auxin response factor 10 [*Carica papaya*] |
| 13 | cpa-miR160d | evm.model.supercontig_49.122\|pacid:16421034 | auxin response factor 17 [*Carica papaya*] |
| **miRNAs downregulated and the corresponding genes upregulated in Papaya** | | | |
| 1 | cpa-miR396 | evm.model.supercontig_43.138\|pacid:16419949 | LOW QUALITY PROTEIN: growth-regulating factor 4-like [*Carica papaya*] |
| 2 | cpa-miR396 | evm.model.supercontig_5.63\|pacid:16421514 | Growth-regulating factor 3 isoform X2 [*Carica papaya*] |
| 3 | cpa-miR396 | evm.model.supercontig_3.145\|pacid:16416419 | Growth-regulating factor 7 [*Carica papaya*] |
| 4 | cpa-miR396 | evm.model.supercontig_28.61\|pacid:16415958 | Growth-regulating factor 1 isoform X3 [*Carica papaya*] |
| 5 | cpa-miR396 | evm.model.supercontig_15.135\|pacid:16409350 | Growth-regulating factor 8-like [*Carica papaya*] |
| 6 | cpa-miR8140 | evm.model.supercontig_119.83\|pacid:16406604 | E3 ubiquitin-protein ligase BRE1-like 2 isoform X4 [*Carica papaya*] |
| 7 | cpa-miR160c/f | evm.model.supercontig_53.88\|pacid:16422316 | Auxin response factor 16-like [*Carica papaya*] |
| 8 | cpa-miR160c/f | evm.model.supercontig_65.4\|pacid:16424448 | LOW QUALITY PROTEIN: auxin response factor 10 [*Carica papaya*] |
| 9 | cpa-miR160c/f | evm.model.supercontig_49.122\|pacid:16421034 | Auxin response factor 17 [*Carica papaya*] |
| 10 | cpa-miR164b | evm.model.supercontig_111.22\|pacid:16405845 | NAC domain-containing protein 100 [*Carica papaya*] |
| 11 | cpa-miR164b | evm.model.supercontig_10.25\|pacid:16404615 | NAC domain-containing protein 21/22-like [*Carica papaya*] |
| 12 | cpa-miR164b | evm.model.supercontig_9.262\|pacid:16428160 | UDP-glucuronic acid decarboxylase 2-like [*Carica papaya*] |
| 13 | cpa-miR164b | evm.model.supercontig_14.101\|pacid:16408594 | NAC domain-containing protein 100-like [*Carica papaya*] |
| 14 | cpa-miR393 | evm.model.supercontig_9.171\|pacid:16428059 | Protein TRANSPORT INHIBITOR RESPONSE 1 [*Carica papaya*] |
| 15 | cpa-miR393 | evm.model.supercontig_70.88\|pacid:16425377 | Protein AUXIN SIGNALING F-BOX 2-like [*Carica papaya*] |
| 16 | cpa-miR156a/b/c | evm.model.supercontig_149.4\|pacid:16409298 | Squamosa promoter-binding-like protein 7 [*Carica papaya*] |
| 17 | cpa-miR156a/b/c | evm.model.supercontig_43.63\|pacid:16420023 | Squamosa promoter-binding-like protein 2 [*Carica papaya*] |
| 18 | cpa-miR156a/b/c | evm.model.supercontig_1095.1\|pacid:16405577 | Uncharacterized protein LOC110813355 [*Carica papaya*] |
| 19 | cpa-miR156a/b/c | evm.model.supercontig_30.96\|pacid:16417050 | F-box/kelch-repeat protein At3g23880-like [*Carica papaya*] |
| 20 | cpa-miR171d | evm.model.supercontig_49.157\|pacid:16421072 | Scarecrow-like protein 6 [*Carica papaya*] |
| 21 | cpa-miR171d | evm.model.supercontig_29.139\|pacid:16416165 | Scarecrow-like protein 22 isoform X1 [*Carica papaya*] |
| 22 | cpa-miR171d | evm.model.supercontig_158.5\|pacid:16409890 | LOW QUALITY PROTEIN: scarecrow-like protein 15 [*Carica papaya*] |
| 23 | cpa-miR535 | evm.model.supercontig_20.9\|pacid:16413119 | KH domain-containing protein HEN4 isoform X1 [*Carica papaya*] |
