# Supplementary material for: Differential expression of microRNAs in response to Papaya ringspot virus infection in differentially responding genotypes of papaya (Carica papaya L.) and its wild relative
Source: Front Plant Sci. 2024 Jun 20;15:1398437. doi: 10.3389/fpls.2024.1398437 (PMC11222417; doi:10.3389/fpls.2024.1398437)
Supplement: Supplementary file 4 [file Presentation_1.pptx]

## Slide 1
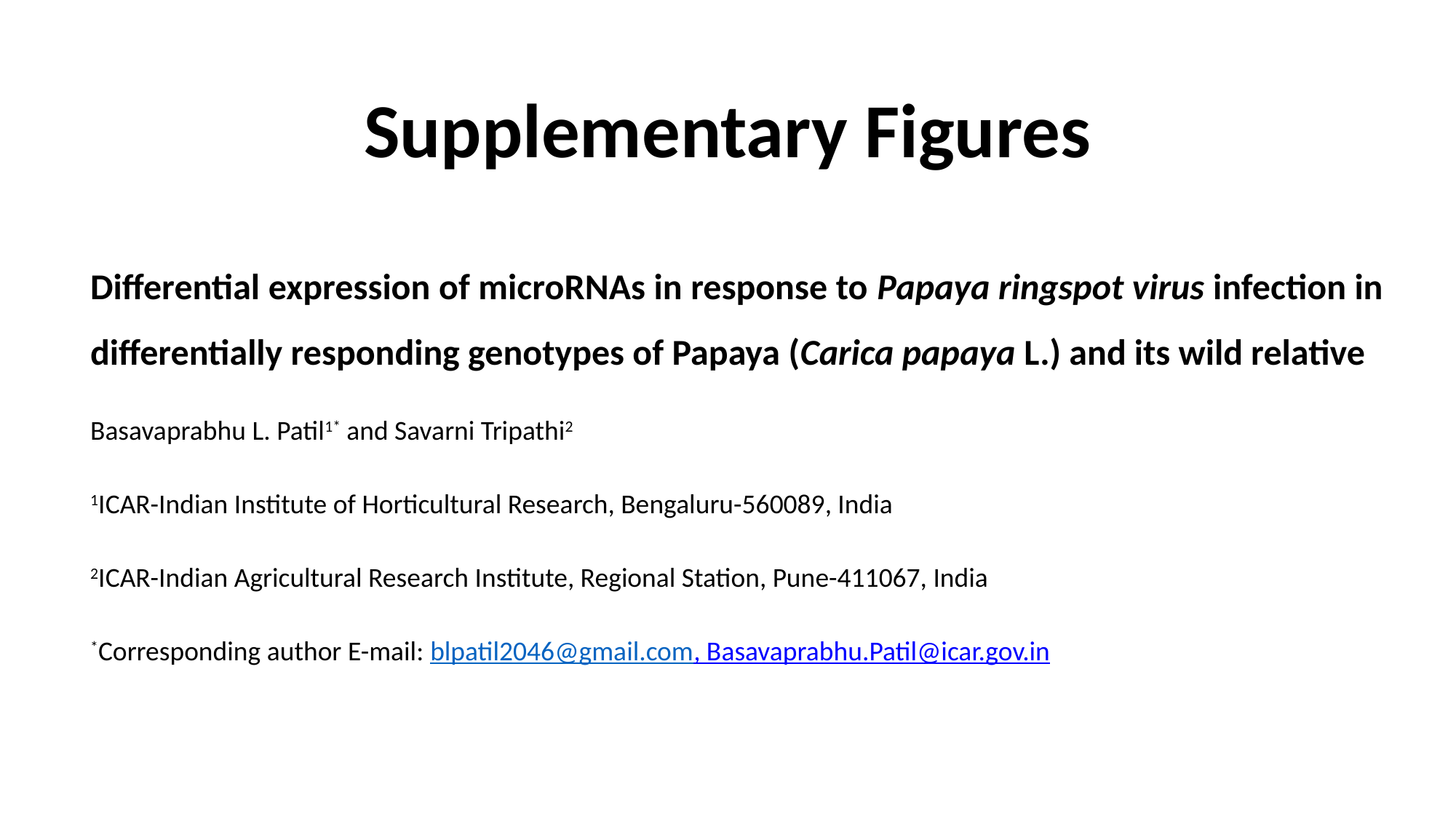

# Supplementary Figures
Differential expression of microRNAs in response to Papaya ringspot virus infection in differentially responding genotypes of Papaya (Carica papaya L.) and its wild relative
Basavaprabhu L. Patil1* and Savarni Tripathi2
1ICAR-Indian Institute of Horticultural Research, Bengaluru-560089, India
2ICAR-Indian Agricultural Research Institute, Regional Station, Pune-411067, India
*Corresponding author E-mail: blpatil2046@gmail.com, Basavaprabhu.Patil@icar.gov.in

## Slide 2
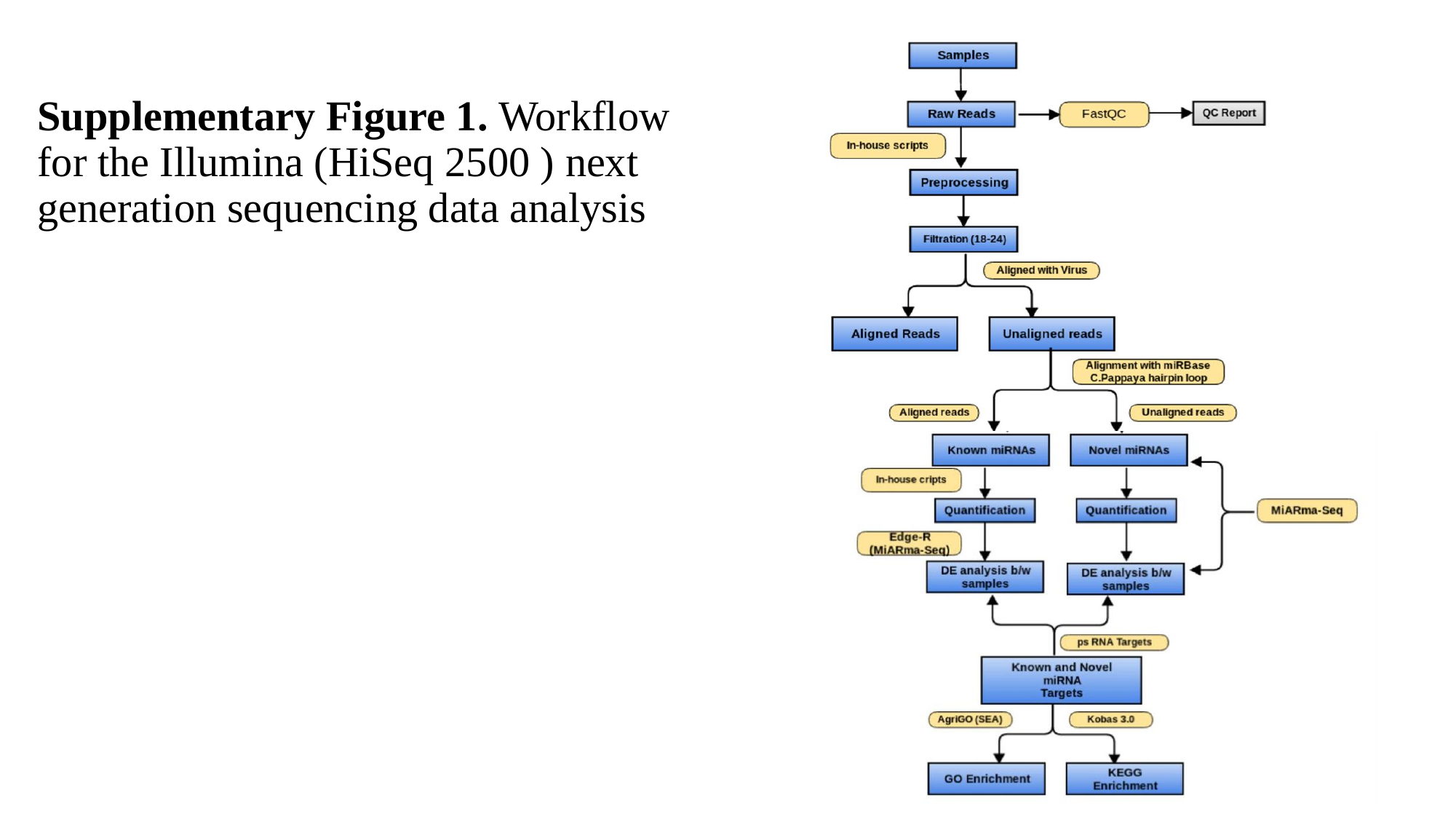

# Supplementary Figure 1. Workflow for the Illumina (HiSeq 2500 ) next generation sequencing data analysis

## Slide 3
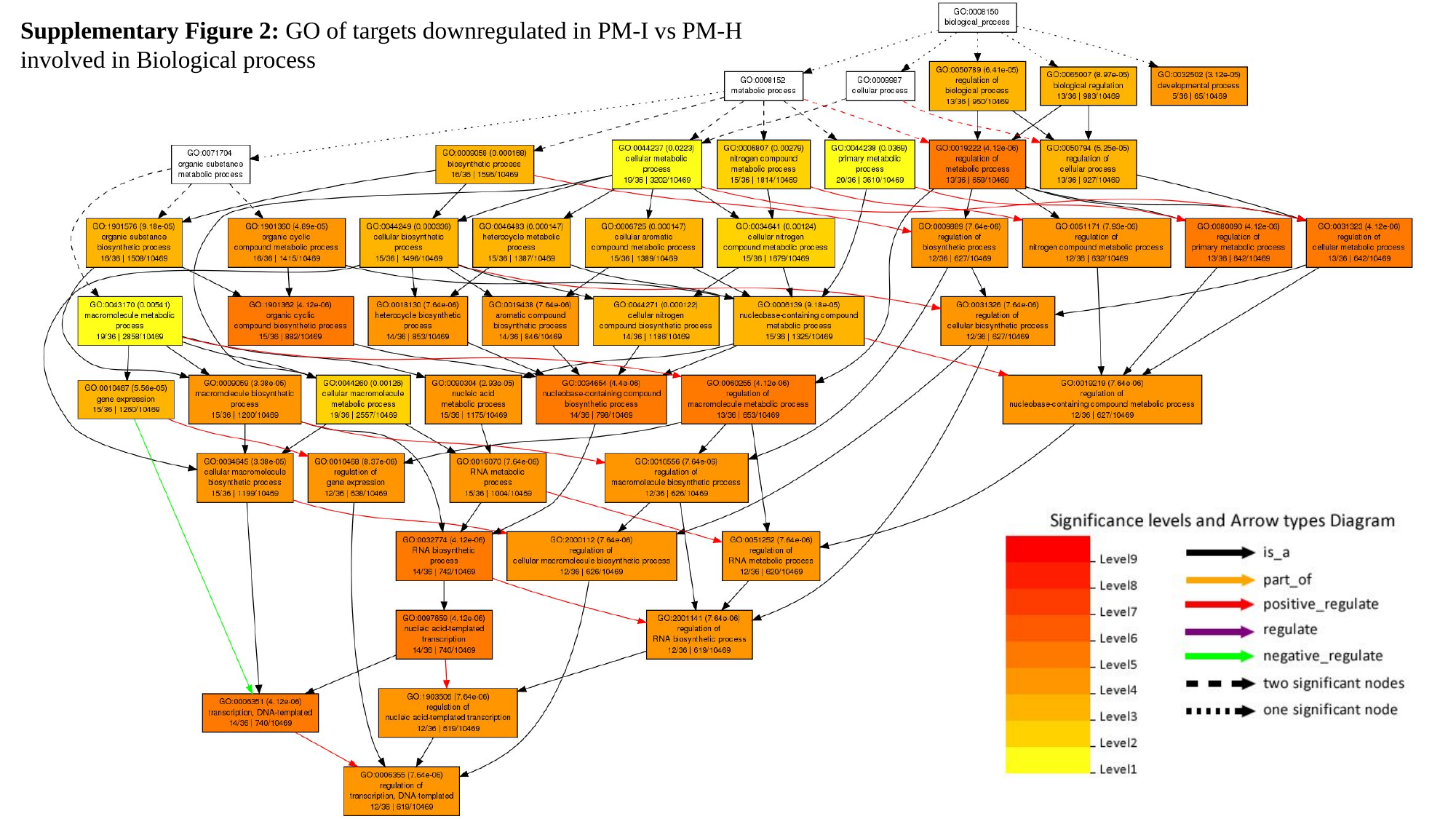

Supplementary Figure 2: GO of targets downregulated in PM-I vs PM-H
involved in Biological process

## Slide 4
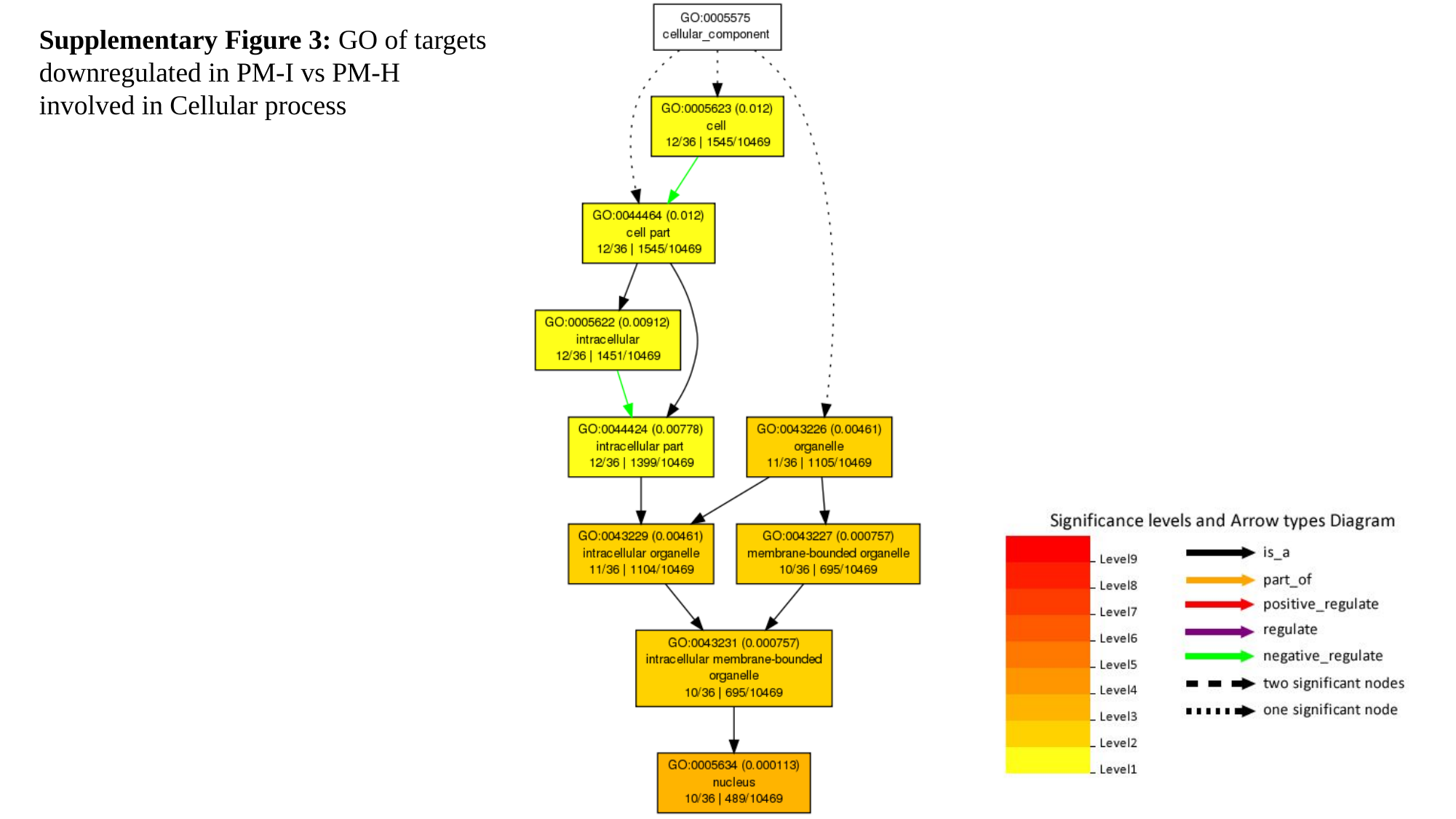

Supplementary Figure 3: GO of targets
downregulated in PM-I vs PM-H
involved in Cellular process

## Slide 5
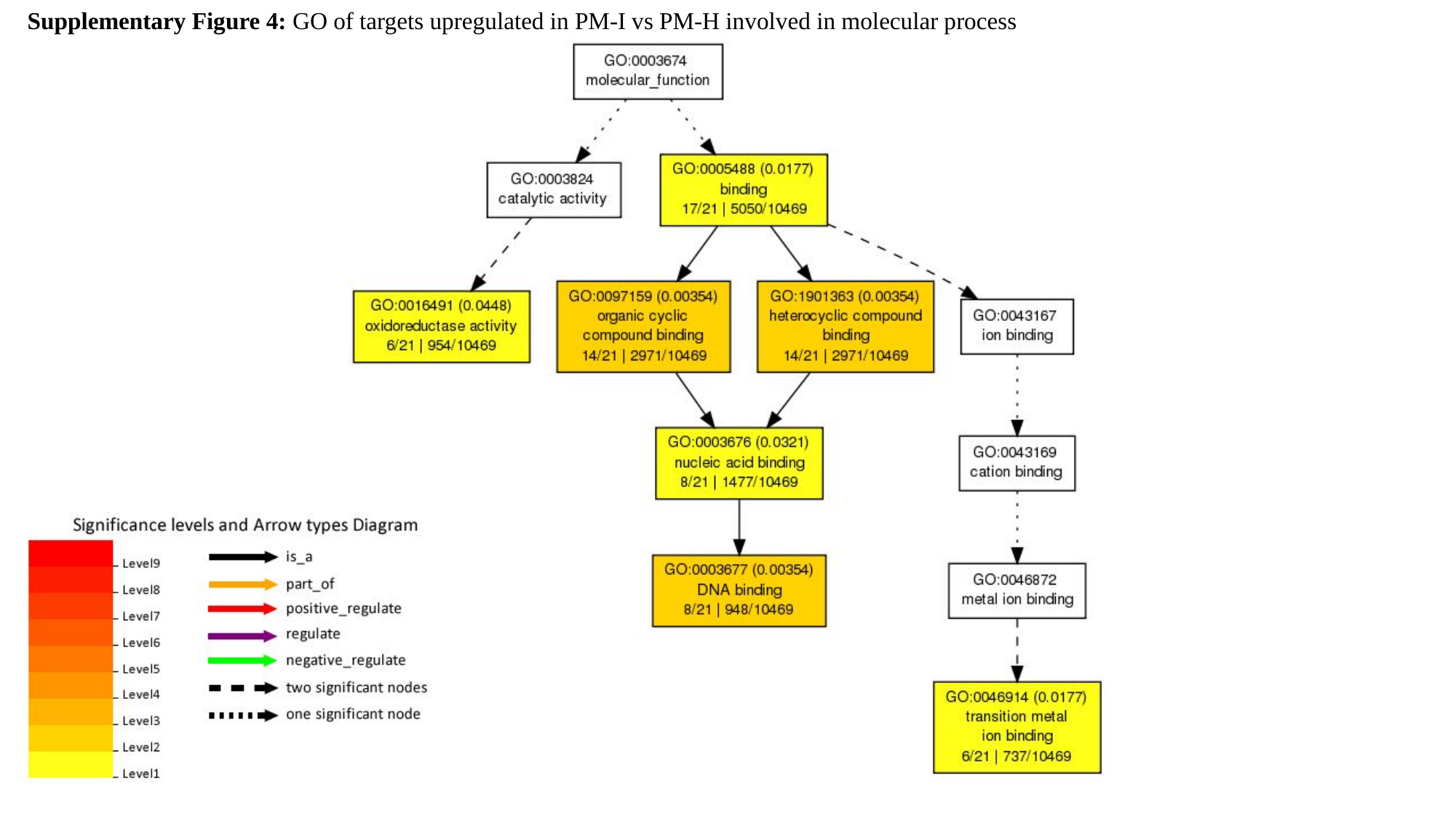

Supplementary Figure 4: GO of targets upregulated in PM-I vs PM-H involved in molecular process

## Slide 6
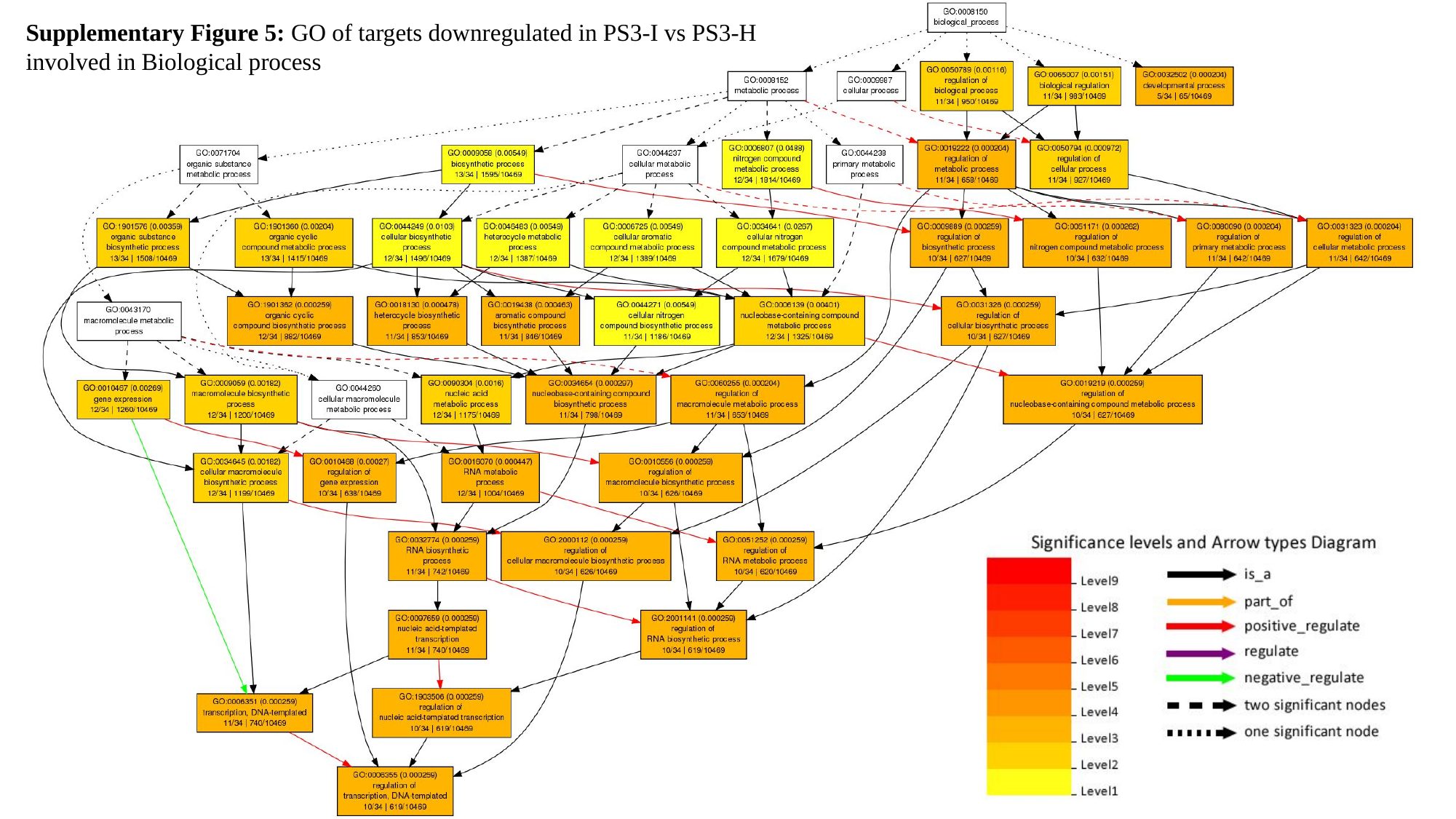

Supplementary Figure 5: GO of targets downregulated in PS3-I vs PS3-H
involved in Biological process
PS3-I vs PS3-H targets downregulated

## Slide 7
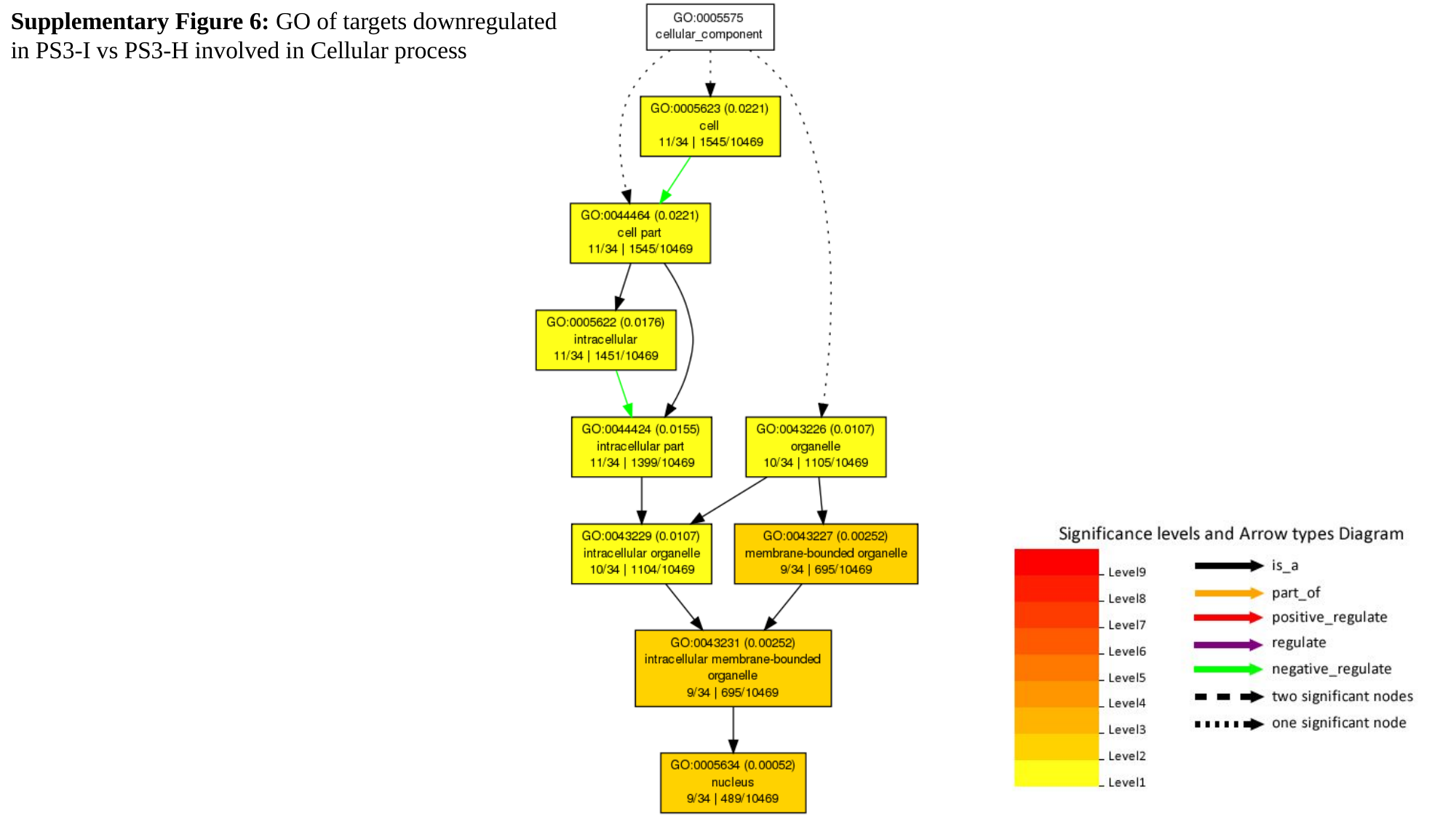

Supplementary Figure 6: GO of targets downregulated
in PS3-I vs PS3-H involved in Cellular process

## Slide 8
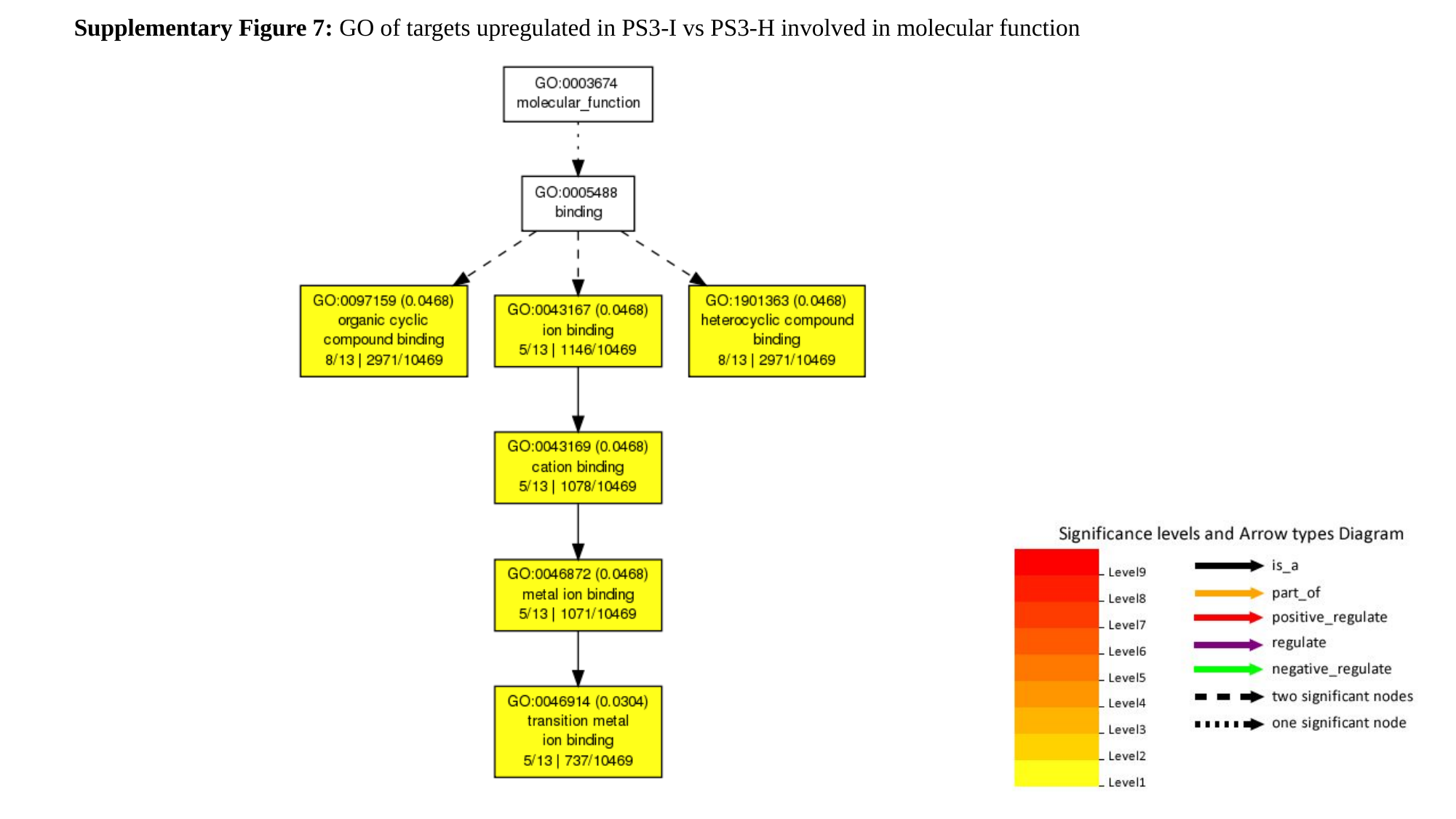

Supplementary Figure 7: GO of targets upregulated in PS3-I vs PS3-H involved in molecular function

## Slide 9
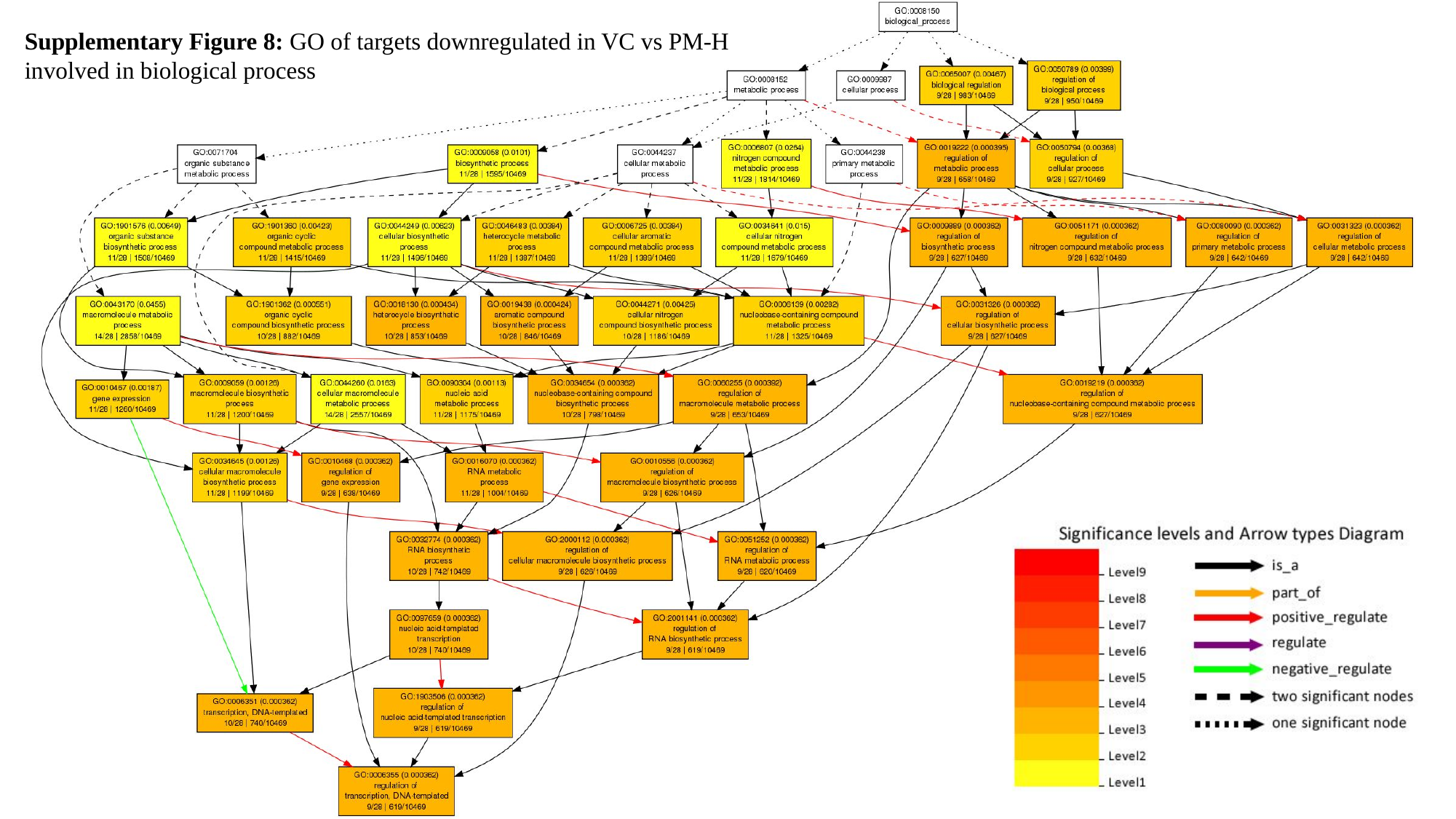

Supplementary Figure 8: GO of targets downregulated in VC vs PM-H
involved in biological process

## Slide 10
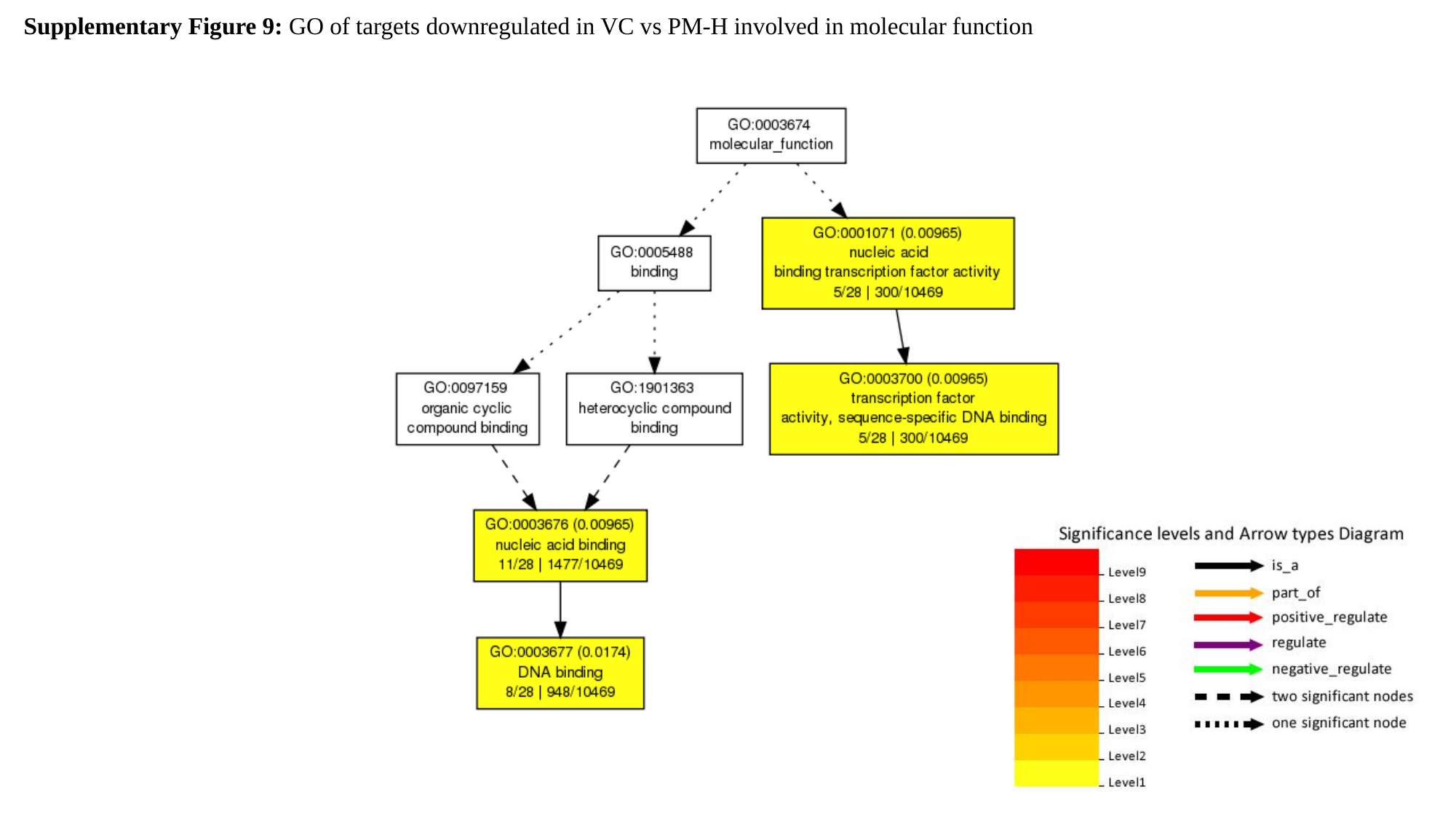

Supplementary Figure 9: GO of targets downregulated in VC vs PM-H involved in molecular function

## Slide 11
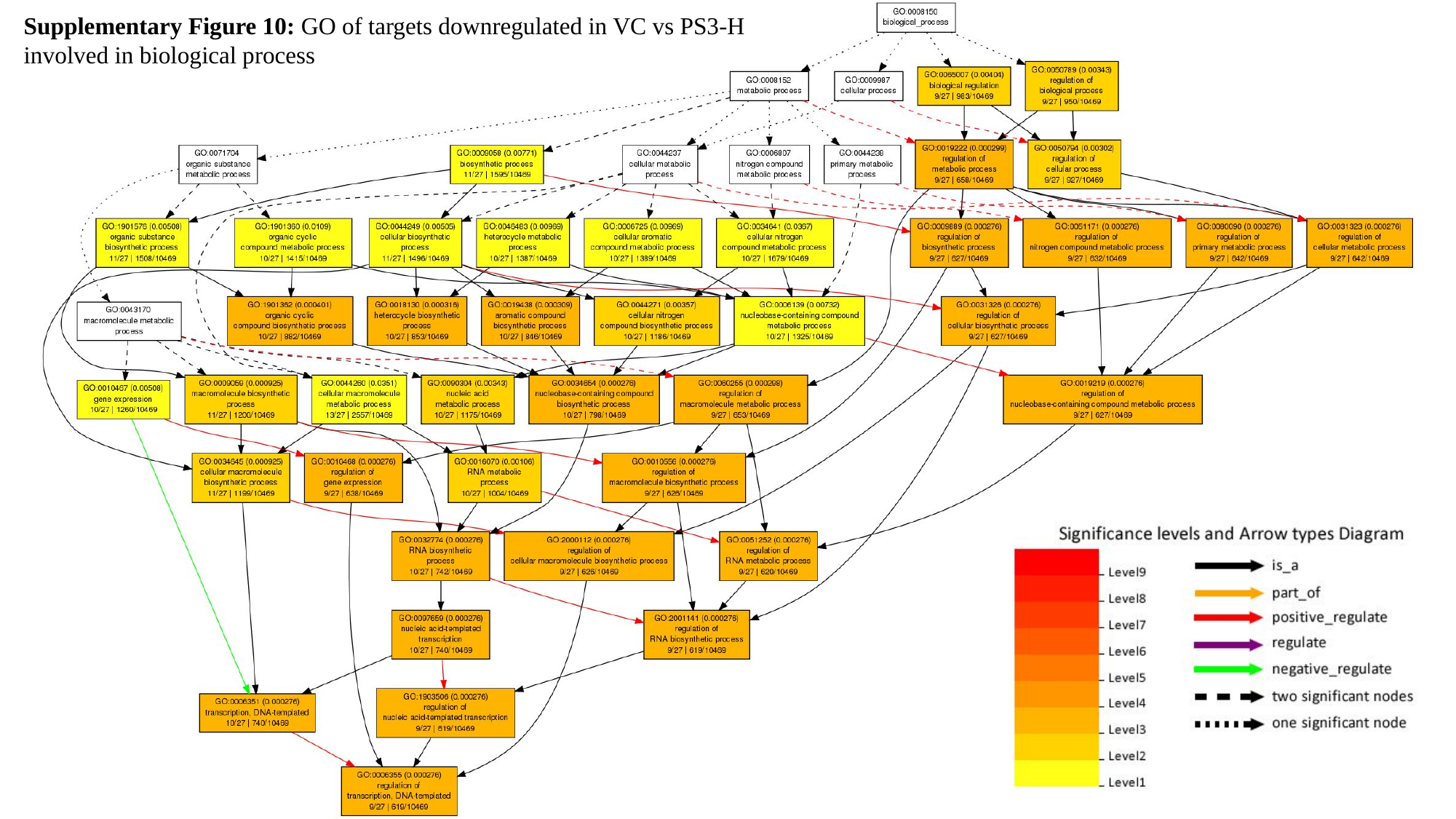

Supplementary Figure 10: GO of targets downregulated in VC vs PS3-H
involved in biological process

## Slide 12
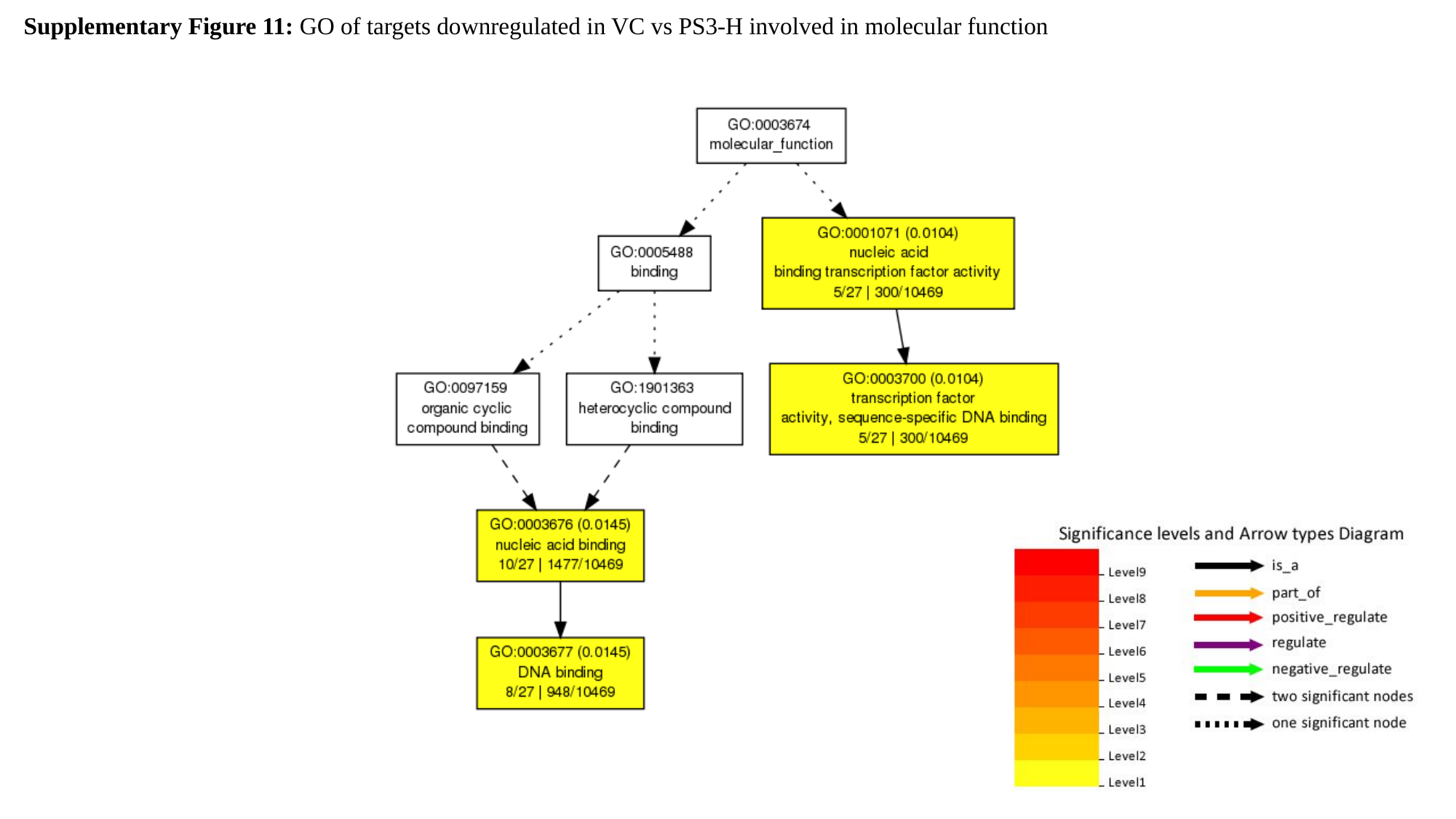

Supplementary Figure 11: GO of targets downregulated in VC vs PS3-H involved in molecular function

## Slide 13
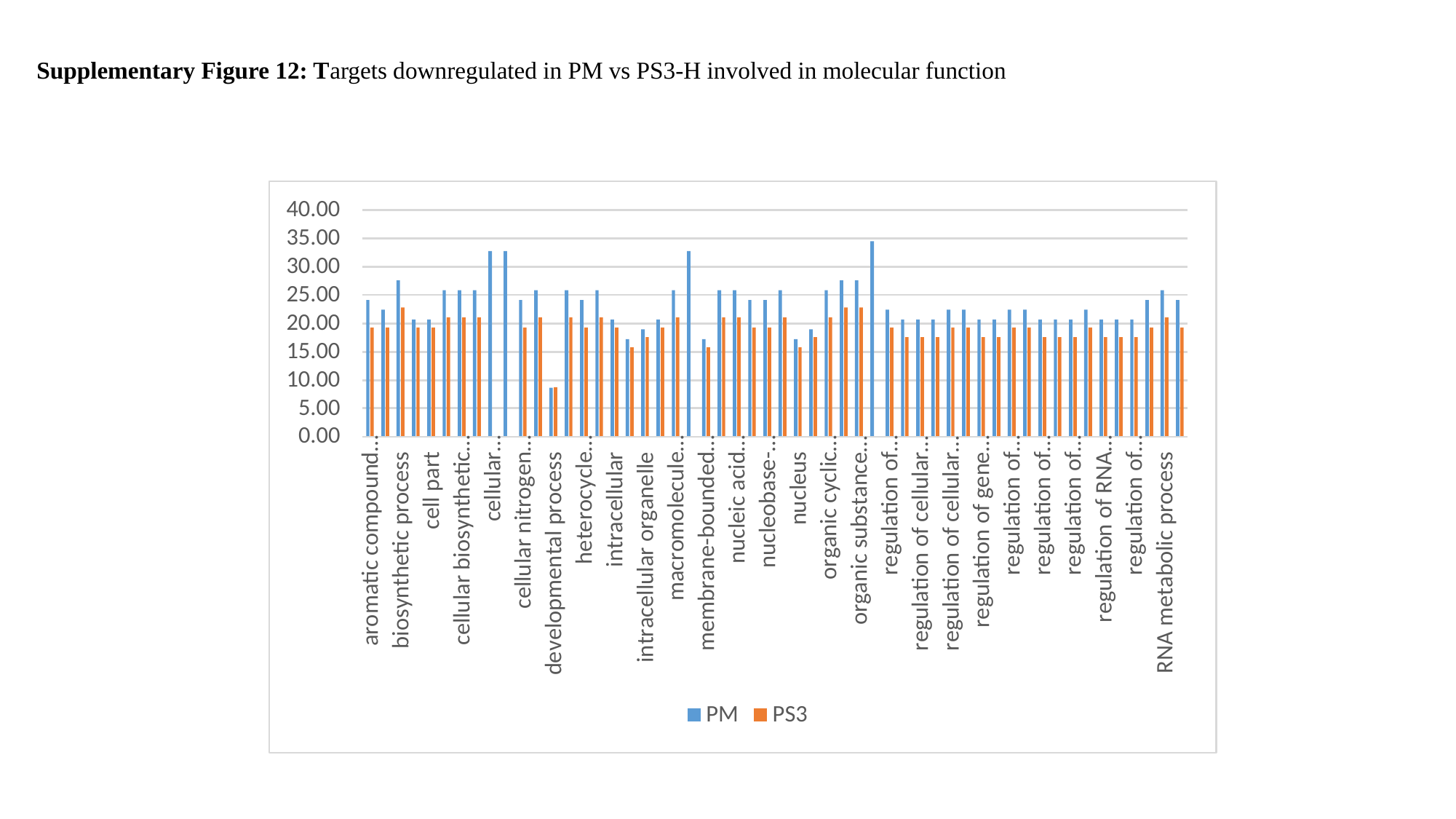

Supplementary Figure 12: Targets downregulated in PM vs PS3-H involved in molecular function

## Slide 14
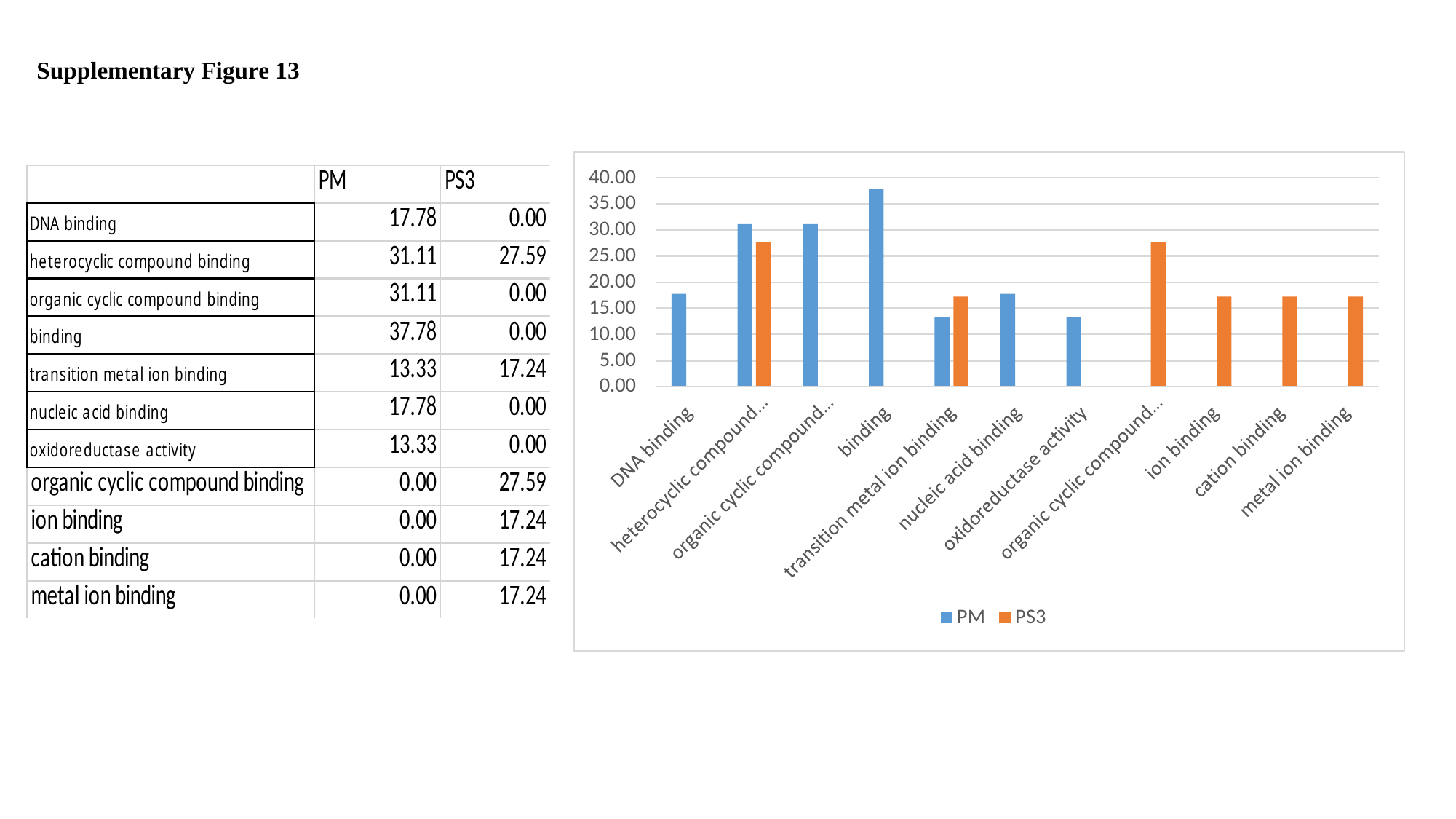

Supplementary Figure 13
