# Supplementary material for: Differential expression of microRNAs in response to Papaya ringspot virus infection in differentially responding genotypes of papaya (Carica papaya L.) and its wild relative
Source: Front Plant Sci. 2024 Jun 20;15:1398437. doi: 10.3389/fpls.2024.1398437 (PMC11222417; doi:10.3389/fpls.2024.1398437)
Supplement: Supplementary file 5 [file Presentation_2.pptx]

## Slide 1
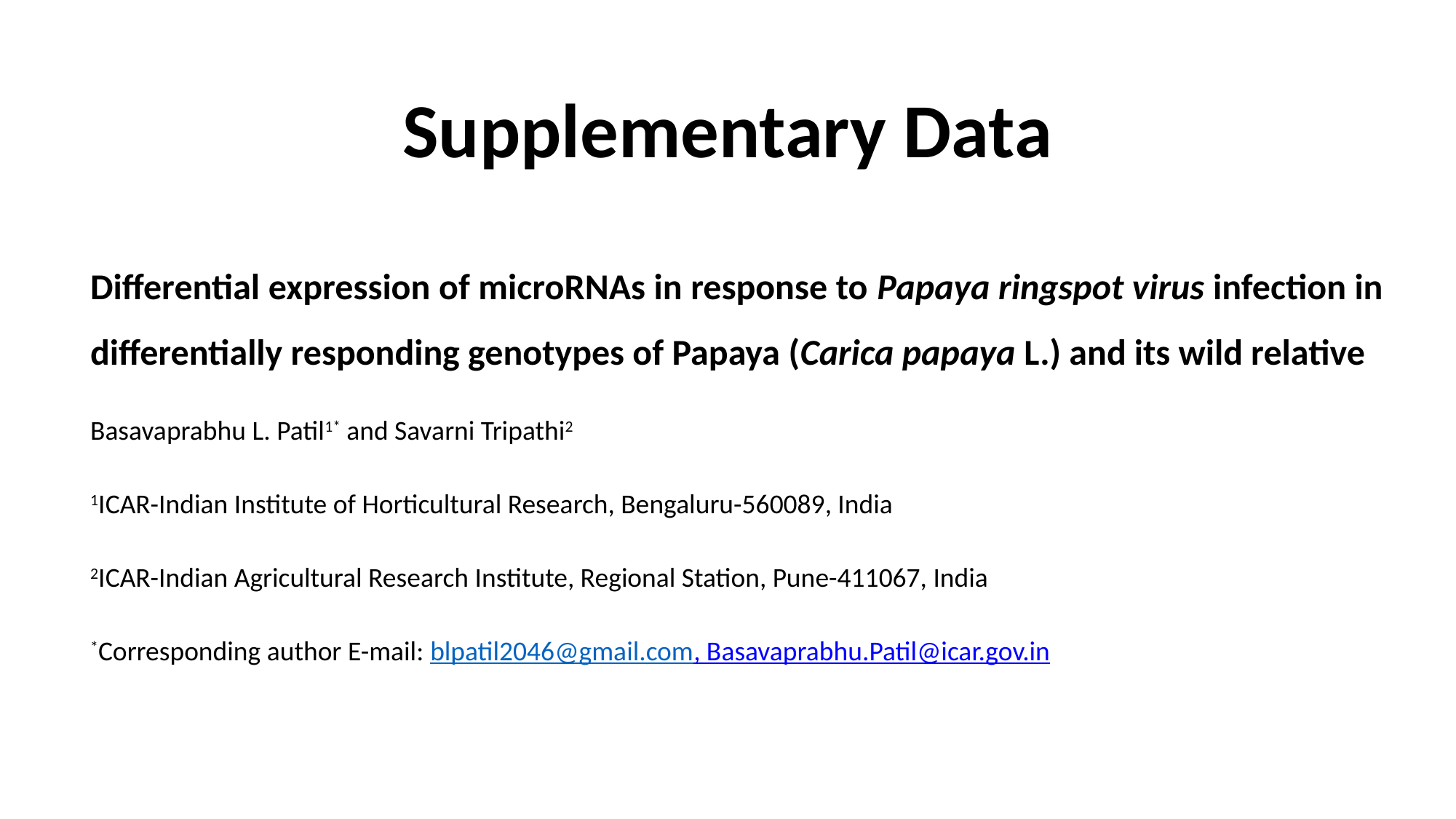

# Supplementary Data
Differential expression of microRNAs in response to Papaya ringspot virus infection in differentially responding genotypes of Papaya (Carica papaya L.) and its wild relative
Basavaprabhu L. Patil1* and Savarni Tripathi2
1ICAR-Indian Institute of Horticultural Research, Bengaluru-560089, India
2ICAR-Indian Agricultural Research Institute, Regional Station, Pune-411067, India
*Corresponding author E-mail: blpatil2046@gmail.com, Basavaprabhu.Patil@icar.gov.in

## Slide 2
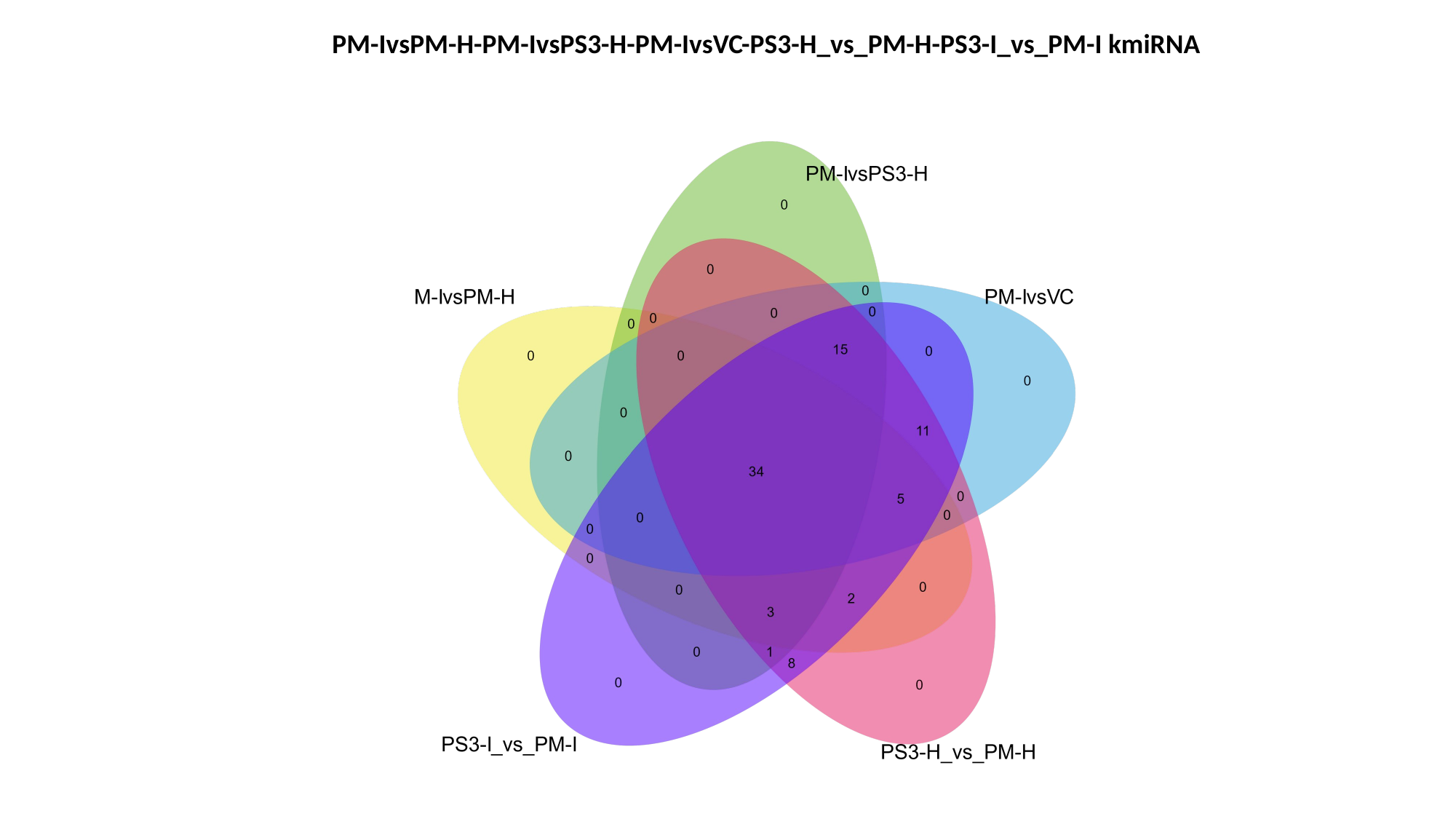

PM-IvsPM-H-PM-IvsPS3-H-PM-IvsVC-PS3-H_vs_PM-H-PS3-I_vs_PM-I kmiRNA

## Slide 3
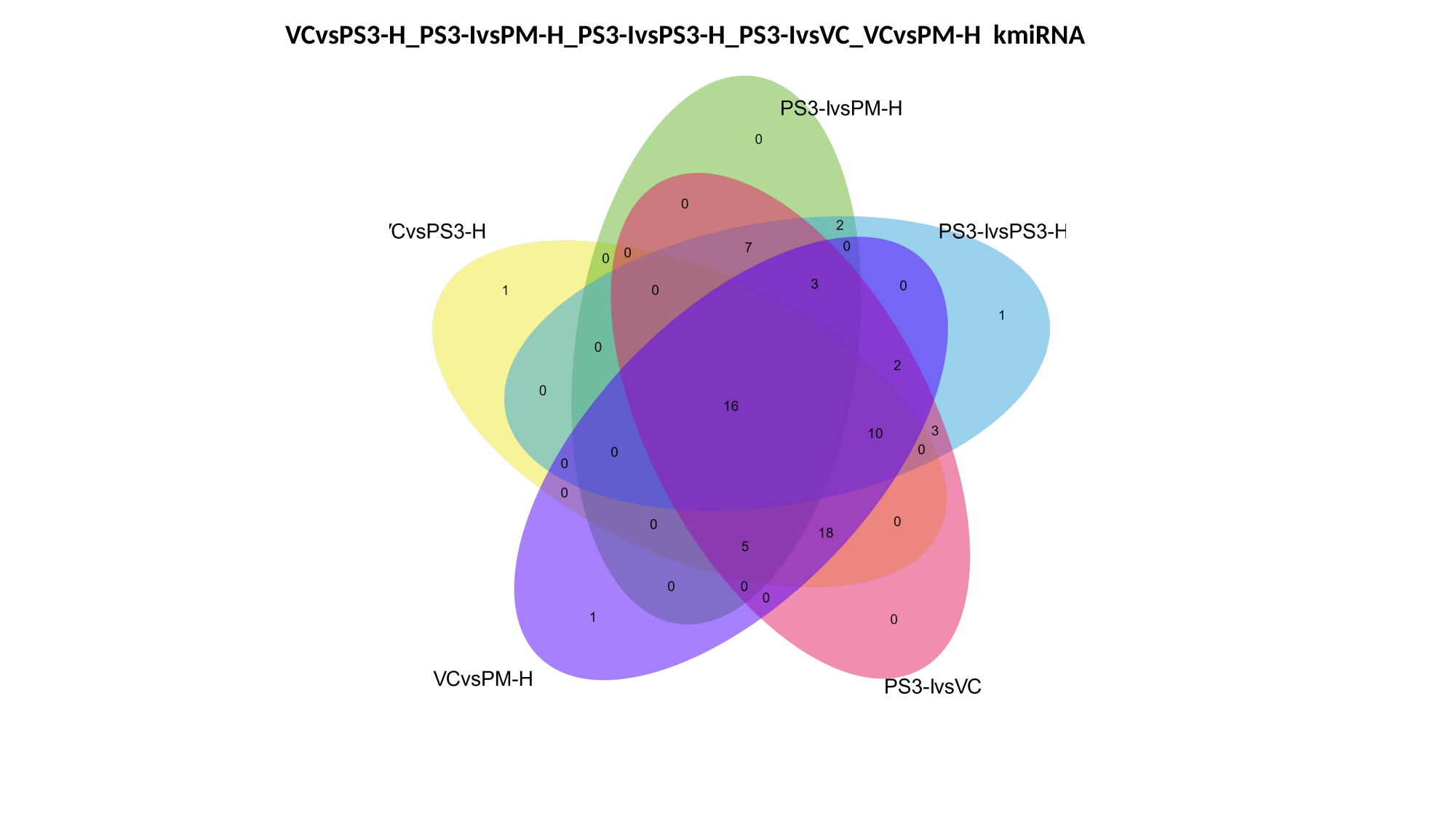

VCvsPS3-H_PS3-IvsPM-H_PS3-IvsPS3-H_PS3-IvsVC_VCvsPM-H kmiRNA

## Slide 4
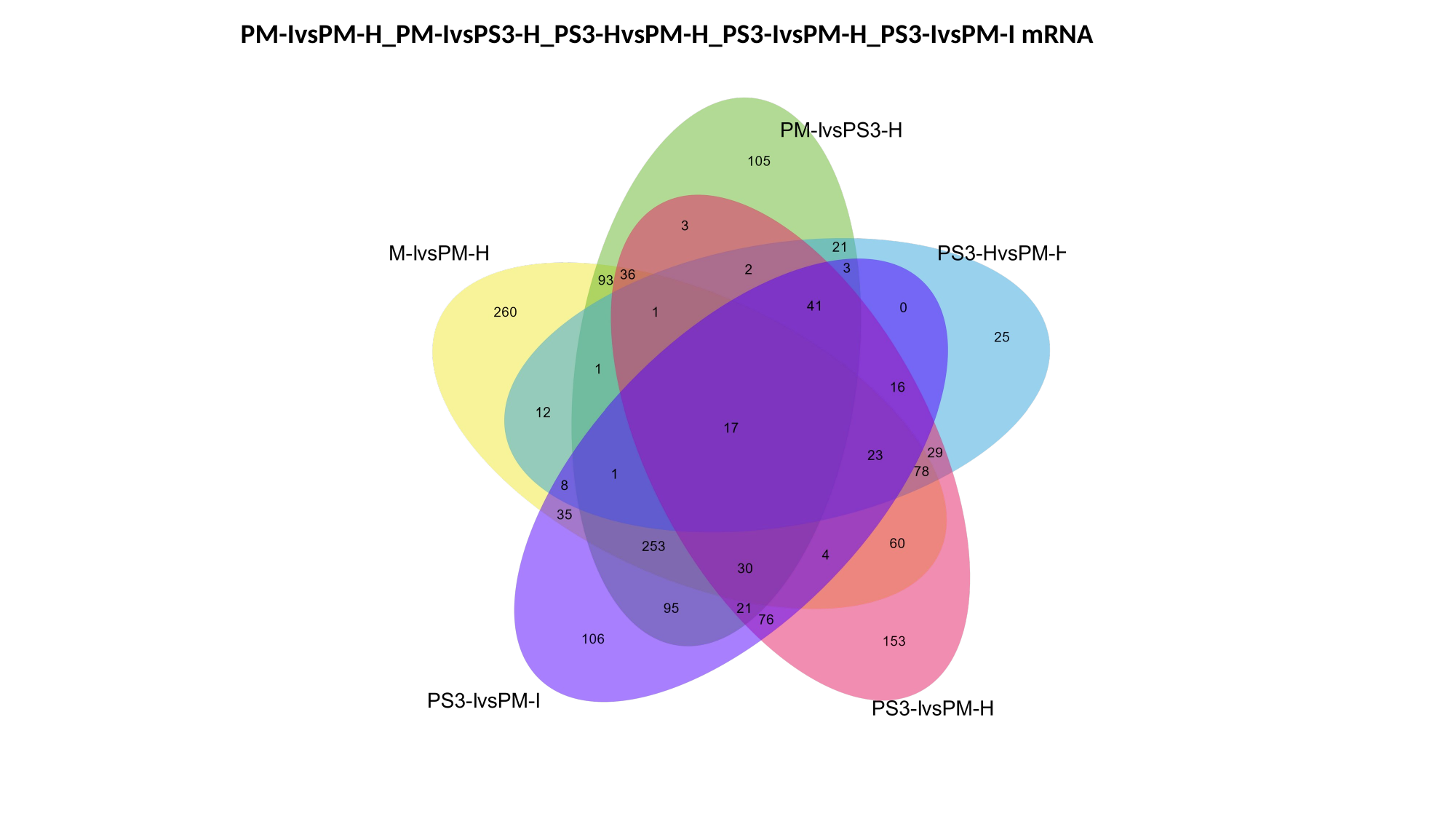

PM-IvsPM-H_PM-IvsPS3-H_PS3-HvsPM-H_PS3-IvsPM-H_PS3-IvsPM-I mRNA

## Slide 5
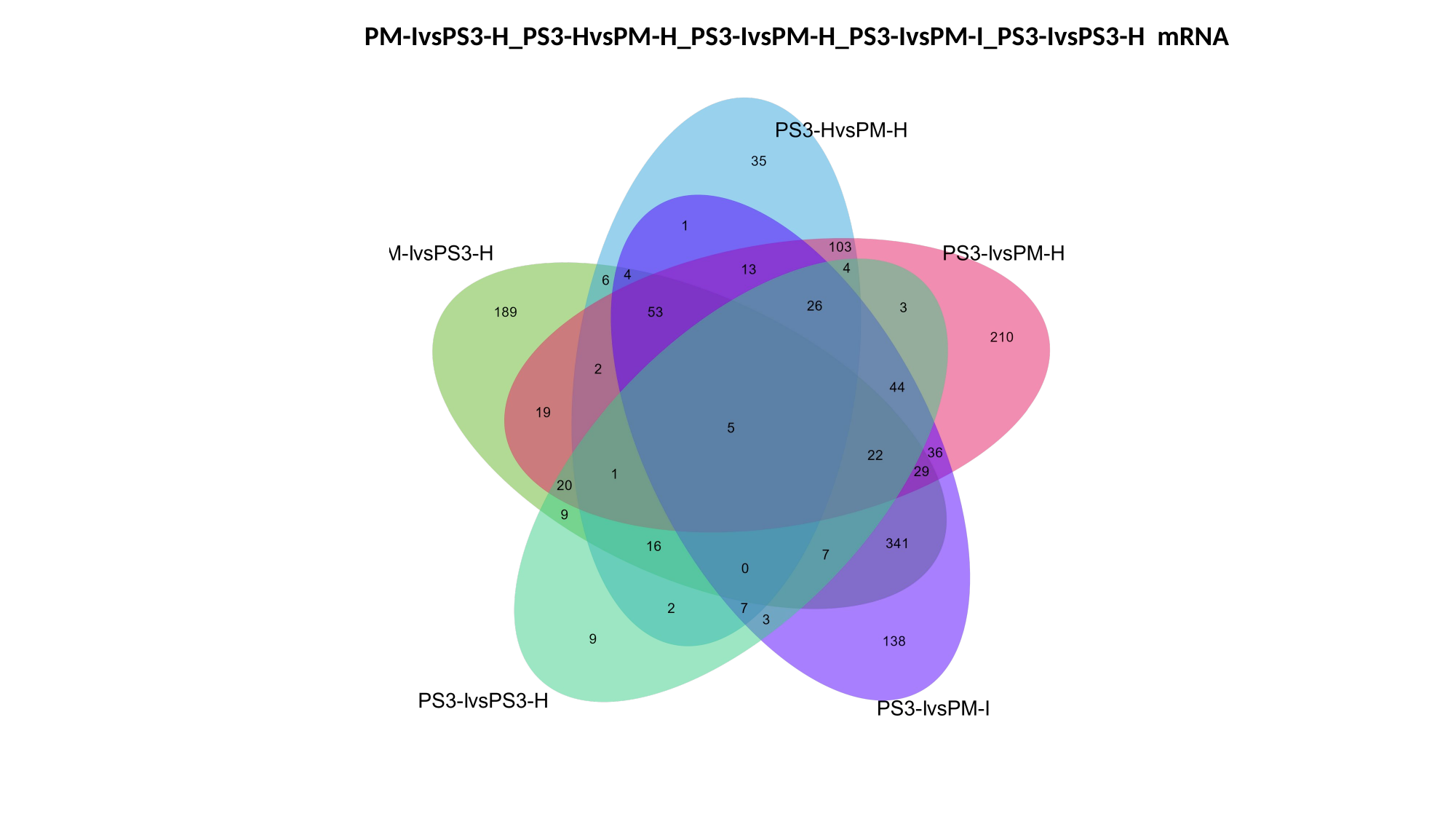

PM-IvsPS3-H_PS3-HvsPM-H_PS3-IvsPM-H_PS3-IvsPM-I_PS3-IvsPS3-H mRNA

## Slide 6
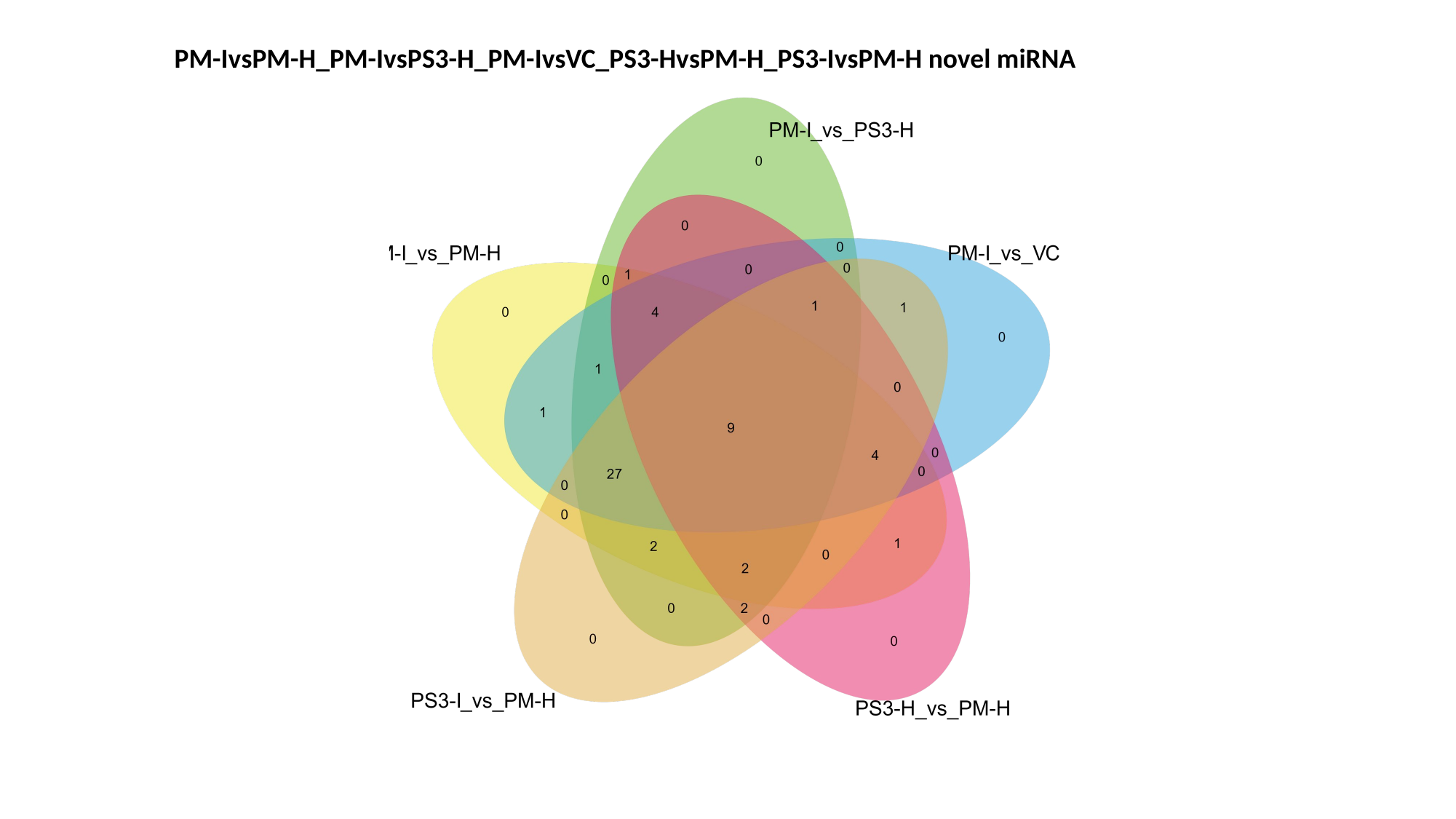

PM-IvsPM-H_PM-IvsPS3-H_PM-IvsVC_PS3-HvsPM-H_PS3-IvsPM-H novel miRNA

## Slide 7
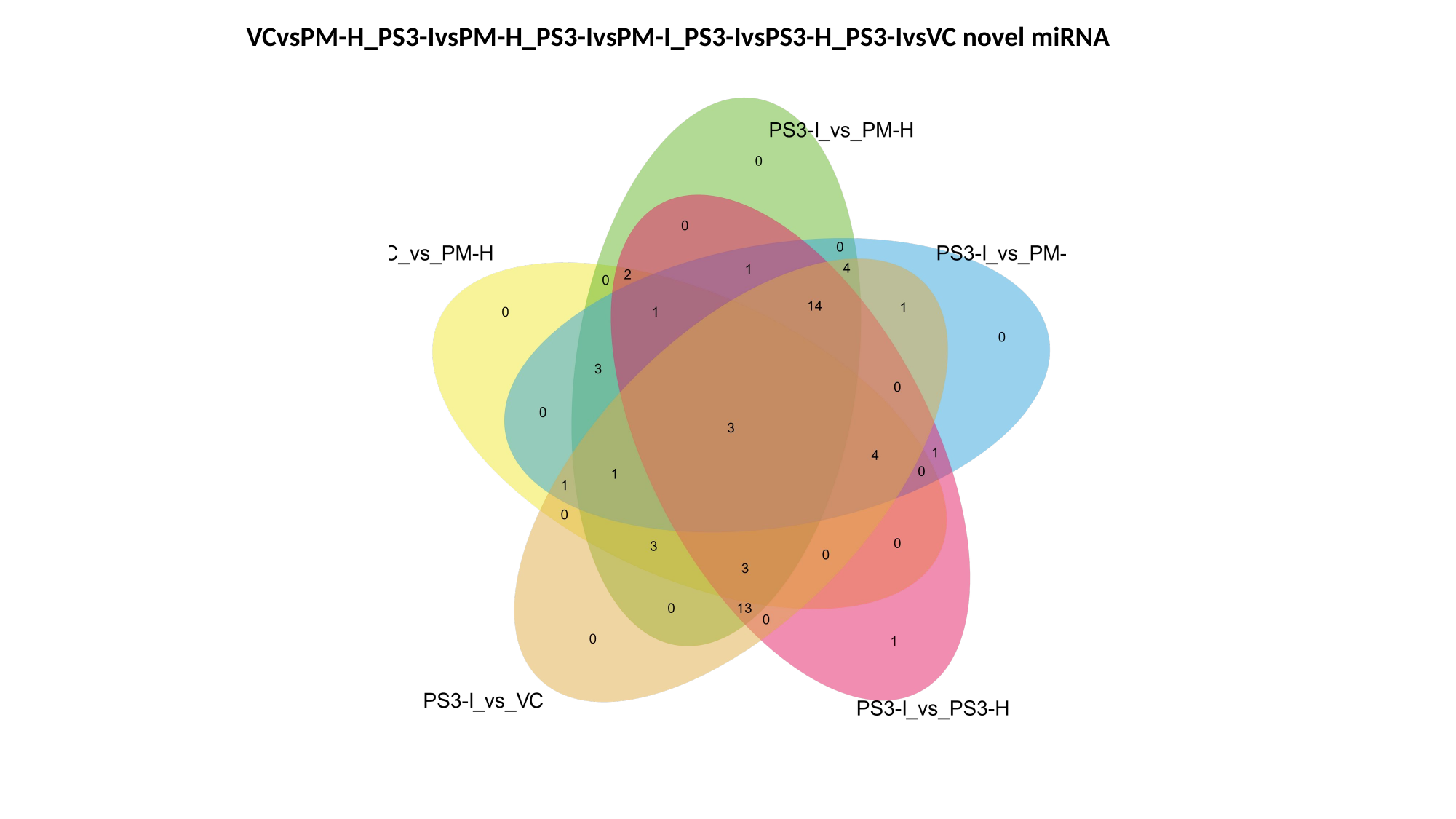

VCvsPM-H_PS3-IvsPM-H_PS3-IvsPM-I_PS3-IvsPS3-H_PS3-IvsVC novel miRNA

## Slide 8
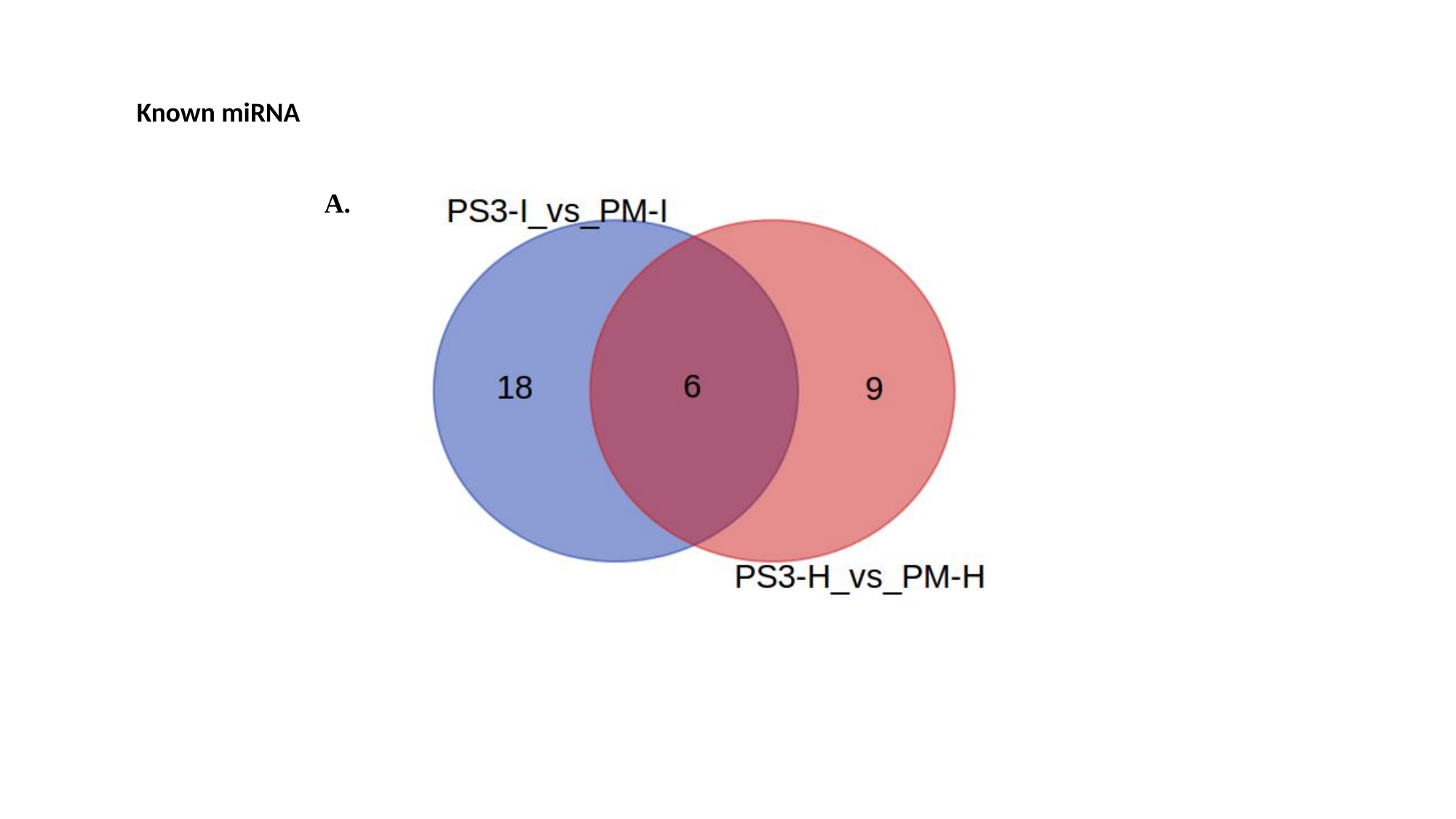

Known miRNA
A.

## Slide 9
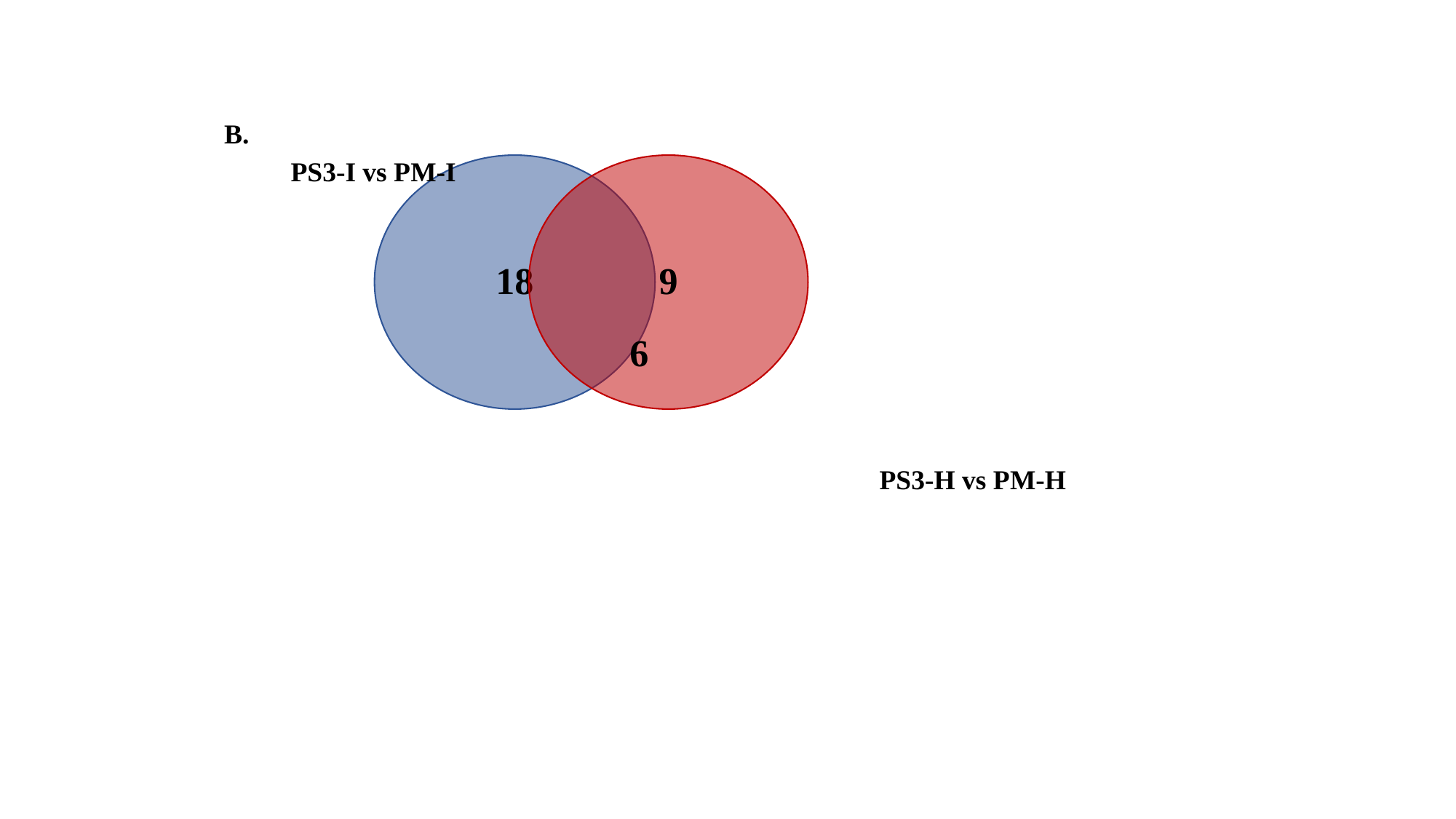

B.
PS3-I vs PM-I
6
PS3-H vs PM-H

## Slide 10
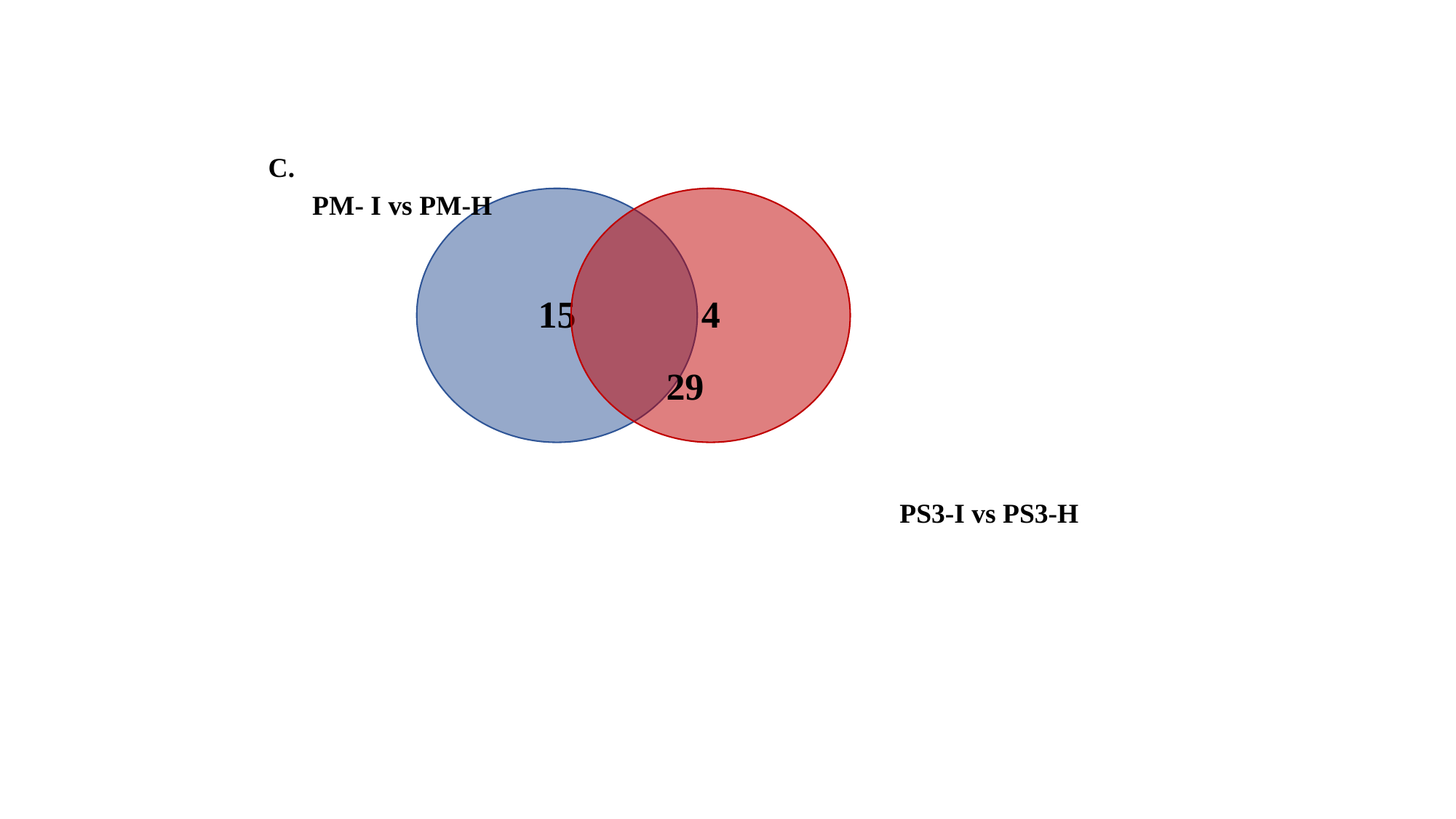

C.
PM- I vs PM-H
29
PS3-I vs PS3-H

## Slide 11
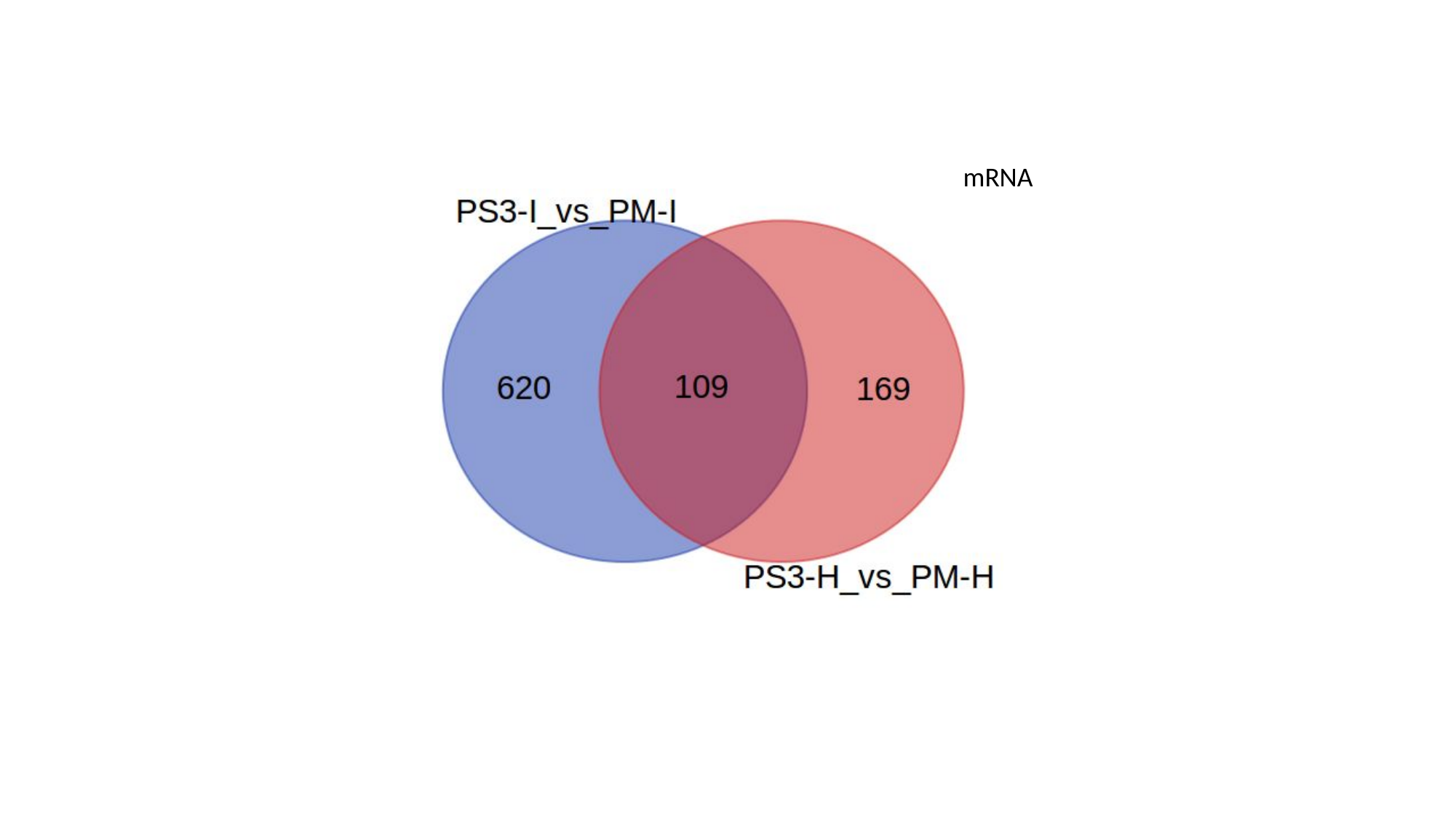

mRNA

## Slide 12
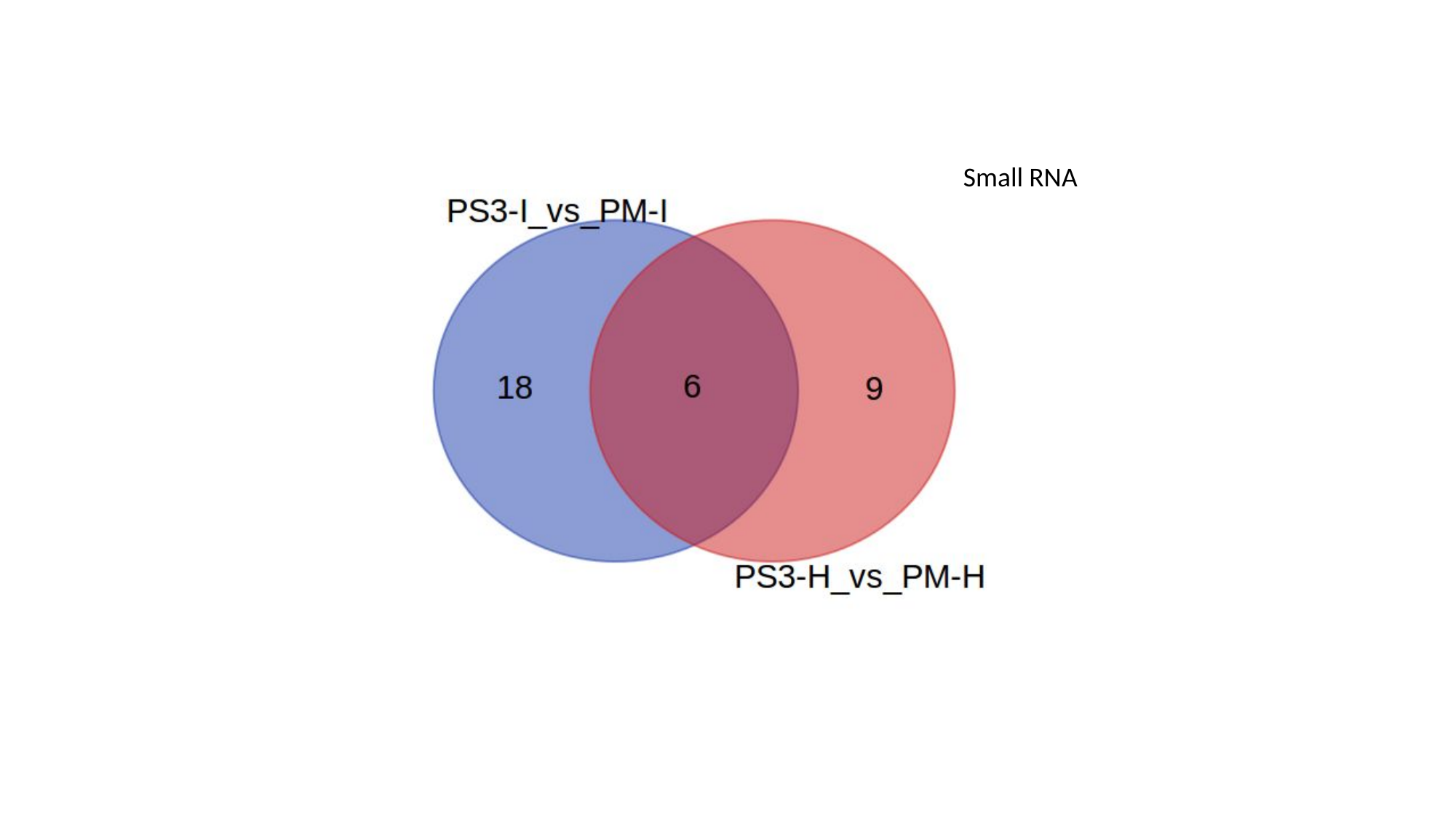

Small RNA
